# Sub‐Unit‐Cell Logic Governs Transport in TPMS Architectures

**DOI:** 10.1002/advs.202523188

**Published:** 2026-03-09

**Authors:** Haozhang Zhong, Yipei He, Jiaxuan Wang, Chenyi Qian, Gerd E Schröder‐Turk, Raj Das, Junye Shi, Qi Tang, Zheda Ning, Wenjue Yi, Chuanwei Li, Jiangping Chen, Zeyao Chen, Binbin Yu, Jian LU, Jianfeng Gu, Ma Qian

**Affiliations:** ^1^ Institute of Materials Modification and Modeling Shanghai Jiao Tong University Shanghai China; ^2^ Centre for Additive Manufacturing School of Engineering RMIT University Melbourne Victoria Australia; ^3^ Institute of Refrigeration and Cryogenics Shanghai Jiao Tong University Shanghai China; ^4^ School of Mathematics, Statistics, Chemistry and Physics Murdoch University Perth Western Australia Australia; ^5^ Research School of Physics The Australian National University Canberra Australian Capital Territory Australia; ^6^ Zhong Guan Cun Research Institute Shanghai Jiao Tong University Liyang China; ^7^ Shanghai Key Laboratory of Materials Laser Processing and Modification Shanghai Jiao Tong University Shanghai China; ^8^ School of Electro‐Mechanical Engineering Guangdong University of Technology Guangzhou China; ^9^ Department of Mechanical Engineering City University of Hong Kong Hong Kong China

**Keywords:** 3D printing, sub‐unit cells, TPMS metamaterials, transport function

## Abstract

Next‐generation energy, thermal, and chemical systems require architectures capable of highly efficient transport across multiple length scales. Triply periodic minimal surfaces (TPMS), first conceptualized in 1865, offer inherently scalable geometries with exceptional transport potential, yet mechanistic links between topology and performance have remained elusive. Here we introduce a sub‐unit‐cell conduit framework that governs transport in TPMS architectures. By integrating crystallographic symmetry analysis with Voronoi tessellation, we show that each TPMS can be resolved into a network of identical intrinsic conduits oriented in different directions, with geometry and connectivity uniquely determined by topology. This framework reveals that transport efficiency is determined primarily by two conduit‐scale descriptors—conduit uniformity and conduit spatial density—while conduit surface area and connectivity play secondary roles. Building on these insights, we derive predictive descriptors and a performance quotient that link local conduit geometry to TPMS transport behavior independent of scale and operating conditions. The model identifies Fischer–Koch as a leading topology, which we validate using additively manufactured copper Fischer–Koch TPMS heat exchangers fabricated via green‐laser powder bed fusion. Experiments reveal up to a 156‐fold improvement in heat‐exchange efficiency (quantified by the j/f ratio) for the copper Fischer–Koch TPMS, compared with a conventional baseline, aligning closely with model predictions. This sub‐unit‐cell conduit approach provides a generalizable mechanistic basis for the rational design of high‐performance TPMS‐architected materials across diverse transport applications.

## Graphical abstract

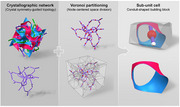

## 1 Introduction

Channel‐based transport architectures underpin essential processes involving mass, heat, and momentum transfer across engineering, chemical, and biomedical systems [1]. Their performance is critical to a variety of applications, including wearable thermal management devices [2], compact heat exchangers [3], microreactors [4], continuous‐flow synthesis platforms [5], artificial lungs [6], and vascular scaffolds [7]. While their design has evolved from simple tubular and plate‐based configurations [8] to sophisticated biomimetic, fractal‐inspired geometries [9, 10], performance remains constrained by persistent efficiency limits, as detailed in Section S1. In thermal transport, for example, most devices operate within a narrow performance band of 0.1–1 for the ratio of heat transfer coefficient (*j*) to pressure drop (*f*)—the *j*/*f* efficiency metric (Table S1) [11].

The recent advent of powder bed fusion (PBF) additive manufacturing (AM) has enabled the fabrication of metallic triply periodic minimal surface (TPMS) architectures, establishing a powerful new metamaterial platform for engineered transport [12, 13, 14, 15, 16, 17]. With their intrinsically intertwined and highly organized channel networks, these architectures offer a promising route to transcend conventional performance limits [18, 19]. Yet this potential remains largely unrealized, owing to the absence of a mechanistic understanding of how transport operates within their labyrinthine networks. As summarized in Table S2, which compiles 50 representative TPMS transport studies published between 2015 and 2025, research in this field began to take shape only around 2015 [12]. To date, studies remain framed at the unit‐cell level [20, 21, 22], relying on averaged geometric metrics, and a fundamental structure‐based predictive or transformative design framework has yet to emerge.

A key challenge is the reliance on unit‐cell‐averaged metrics (Table S1), which characterize TPMS architectures using bulk parameters such as porosity and surface‐area density. Although the unit cell is a fundamental periodic design module, this level of description is too coarse to capture the conduit‐scale geometries that actually govern transport—local variations in channel shape, connectivity, and curvature that control flow and heat exchange. As a result, fundamentally different TPMS topologies can appear indistinguishable under volume‐averaged descriptors, creating a “black‐box” effect that obscures the physical origins of their transport behavior.

Here, we address this limitation by introducing a sub‐unit‐cell conduit framework that decomposes TPMS architectures into their fundamental geometric transport domains. Using symmetry‐guided decomposition and multivariate analysis, we identify how these intrinsic units connect and curve to form continuous, topology‐specific pathways, unveiling a governing sub‐unit‐cell logic for TPMS transport. This logic is validated experimentally through green‐laser PBF (GL‐PBF)–fabricated Fischer–Koch TPMS copper heat exchangers, which achieve a 156‐fold enhancement in j/f efficiency. Collectively, our results establish sub‐unit‐cell architectural control as a principled path toward breaking long‐standing transport‐efficiency limits in TPMS structures.

## 2 Sub‐Unit‐Cell Topology Analysis: Decoding Fundamental Conduit Domains in TPMS

**FIGURE 1:**
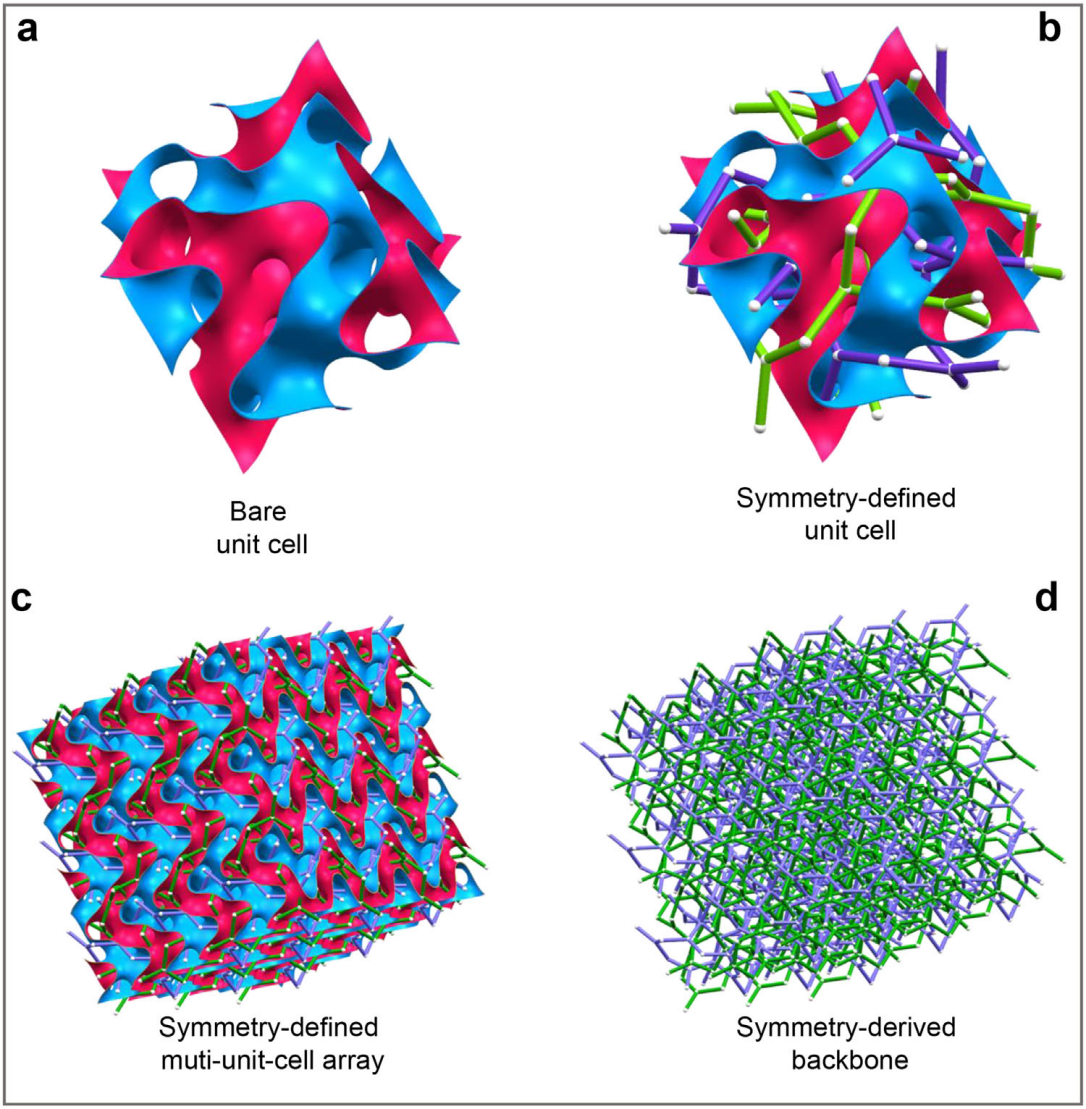
Symmetry‐defined TPMS architecture and its crystallographic skeleton. (a) A unit cell of the Fischer–Koch TPMS. (b) The two interwoven skeletal networks generated by the crystallographic symmetry of space group $I\bar{4}3d$. (c) A 3 × 3 × 3 TPMS lattice array showing the minimal surface and its underlying skeleton, demonstrating how symmetry dictates conduit geometry across the periodic structure. (d) The isolated dual skeletons revealing the intrinsic connectivity and branching topology of the transport network. Table S4 lists six crystallographic symmetry and skeletons of six TPMS configurations.

TPMS geometries arise by partitioning space into two interwoven, mutually disconnected domains, as illustrated by the red and blue regions in Figure 1a. To resolve the intrinsic geometric order underlying this biphasic structure, we introduce a crystallographic‐symmetry–guided dissection framework. For instance, the Fischer–Koch TPMS belongs to the cubic space group $I\bar{4}3d$ (No. 220) [23]. Applying a spatial symmetry analysis tool [24], we extract its underlying crystallographic organization, revealing two interwoven and topologically equivalent skeletal networks that form the structural scaffold of the surface (Figure 1b).

**FIGURE 2:**
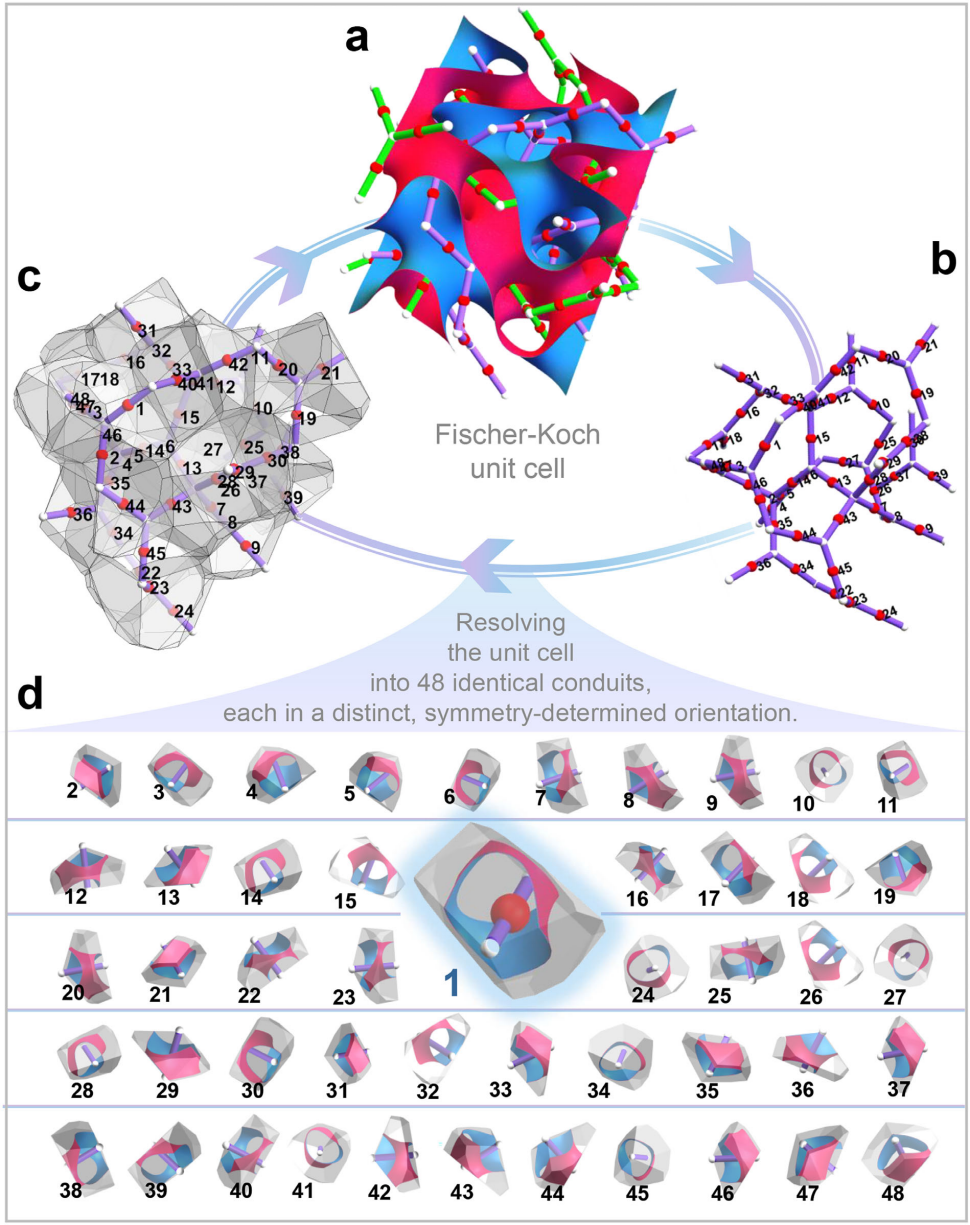
Decoding Fischer‐Koch unit cell into 48 identical conduit‐like subunits differing only in orientation via crystallographic symmetry and Voronoi partitioning. (a) Crystallographic decomposition of the Fischer–Koch TPMS (space group $I\bar{4}3d$, No. 220) reveals two interwoven skeletal networks. (b) A single skeletal network (purple) is selected. The midpoints of its 48 conduit segments (white spheres, labeled 1–48) serve as seed points for the Voronoi tessellation. (c) The resulting Voronoi cells, bounded by the minimal surface, partition the volume into discrete flow conduits. This tessellation retroactively segments the original surface from (a), creating a closed analytical loop (a→b→c→a) that decodes the sub‐unit‐cell transport domains. (d) The complete set of 48 intrinsic conduits—Voronoi‐defined minimal surfaces of conduit shape. The Voronoi‐wrapped minimal surface, the conduit itself, constitutes the identical sub‐unit cell; all 48 differ only in spatial orientation. One conduit (No. 1) is magnified, with the others shown at reduced scale to emphasize uniformity. The defining Voronoi partitions, also geometrically identical and orientation‐varied, are visualized. Further details are in Section S2.

At the level of the extended periodic architecture (Figure 1c–d), this scaffold resolves into distinct families of symmetry‐defined conduit segments—straight, curved, and junction types—that recur throughout the lattice. Figure 1c illustrates the alignment of these symmetry‐dictated pathways with the enclosing minimal surface, while Figure 1d isolates the scaffold to reveal the crystallographic backbone formed by these repeating segments. This backbone provides the structural basis for the sub‐unit‐cell transport domains introduced in Figure 2.

To convert this crystallographic backbone into a set of sub‐unit‐cell transport units, we employ a Voronoi‐based geometric decomposition [25, 26]. First, a single symmetry‐defined skeletal network is selected as the reference scaffold (Figure 2a), and the midpoints of its 48 conduit segments are used as Voronoi seed points (Figure 2b). The resulting tessellation partitions the TPMS volume into discrete, channel‐shaped subdomains bounded by the minimal surface (Figure 2c).

These Voronoi subdomains constitute the fundamental repeating transport units of the TPMS. We define these volumetric, conduit‐shaped regions as *intrinsic conduits*—the smallest geometric domains that physically confine and guide flow. Each Fischer–Koch unit cell contains 48 such identical conduits (Figure 2d), forming a modular architecture from which global transport behavior emerges. This decomposition effectively opens the “unit‐cell black box” by revealing the underlying geometric organization that dictates transport. For clarity, Figure 2d magnifies one representative intrinsic conduit (labeled No. 1), while the remaining 47 are shown at a reduced scale to emphasize their structural uniformity.

It should be emphasized that, for any given TPMS architecture, all intrinsic conduits are geometrically identical and topologically equivalent—an inherent symmetry of TPMS. As illustrated in Figure 2, although the conduits within a unit cell may appear distinct in a fixed orientation, they are in fact identical: each conduit can be rotated to align exactly with any other. Figure S3 further demonstrates this principle by constructing a Fischer–Koch TPMS unit cell step by step from a single conduit. This confirms their fundamental uniformity and validates the conduit as the symmetric sub‐unit of the architecture.

**FIGURE 3:**
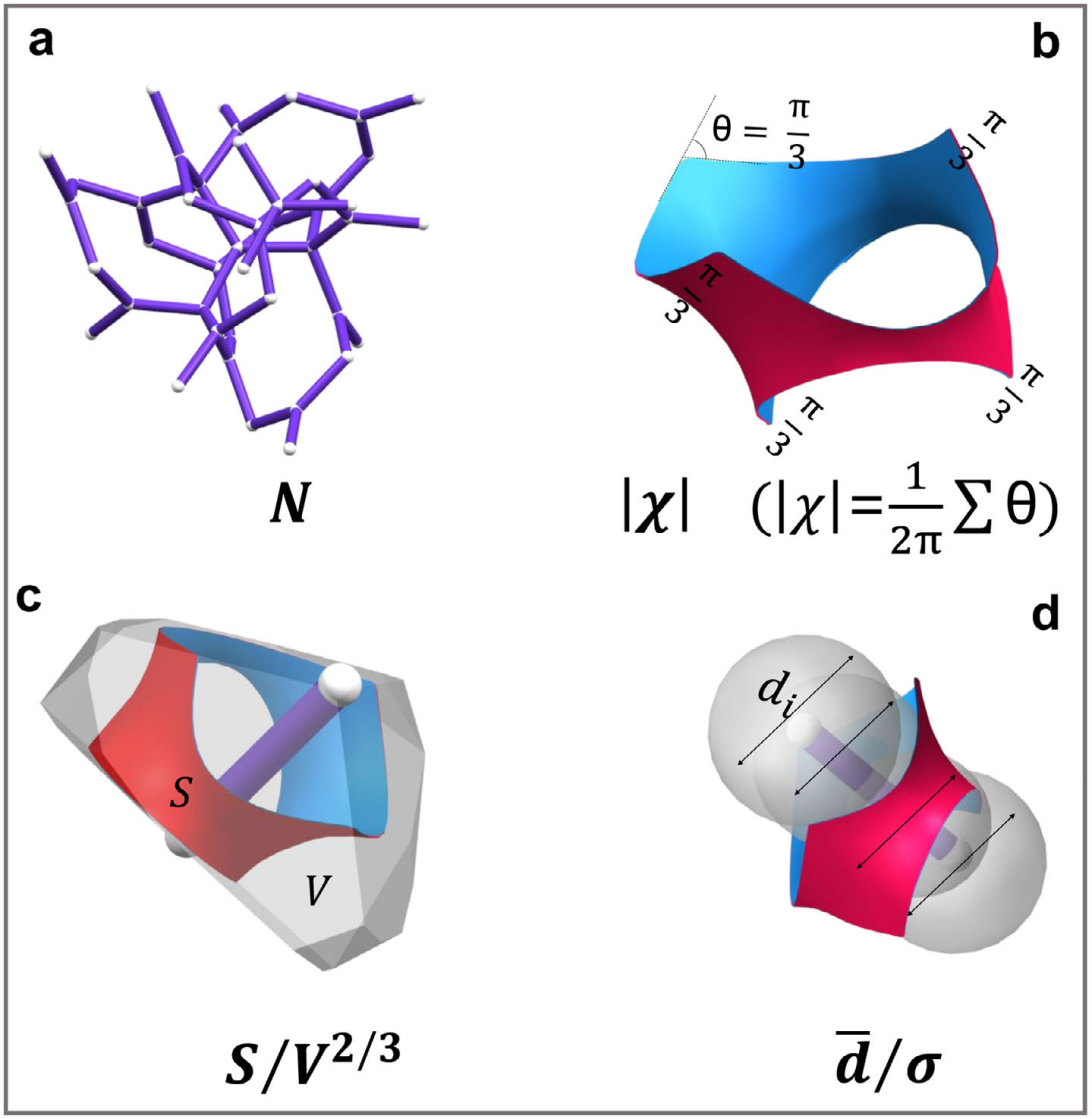
Sub‐unit‐cell geometric and topological descriptors governing intrinsic transport, illustrated using the Fischer–Koch TPMS as a representative case. The two‐channel networks are structurally equivalent; the purple skeletal network is shown here. (a) Spatial density (*N*): The number of intrinsic transport domains (conduits) per unit cell. (b) Connectivity (Euler index, |*χ*|): A measure of topological complexity, calculated via the Gauss‐Bonnet theorem as $\left|\chi \right|=\frac{1}{2\pi }\;\left|\int_{M}\kappa dA\right|$ for smooth surfaces, or equivalently, as $\left|\chi \right|=\frac{1}{2\pi }\;\sum \theta$ for discretized surfaces, where θ denotes the angular deficit (in radians) [32]. For a representative sub‐unit‐cell domain in the Fischer–Koch TPMS, each angular deficit is $\theta =\frac{\pi }{3}$, and five such equivalent angular deficits are present, as shown in Figure 3b. Accordingly, $\Sigma \theta =\frac{\pi }{3}\;\times 5$, yielding |*χ*| =  0.83. (c) Surface utilization (*S*/*V* ^2/3^): *S* is the area of the minimal surface (red or blue), and *V* is the volume of the Voronoi cell. (d) Geometric uniformity $\left(\bar{d}/\sigma \right)$. $\bar{d}$: conduit mean diameter, calculated via Voronoi tessellation, which can well capture the effective hydraulic clearance experienced by the fluid throughout the labyrinthine channels in TPMS. σ: Standard deviation of $\left(\bar{d}/\sigma \right)$. For further details, see Section S2.

To operationalize this framework for transport‐oriented TPMS design, we define four dimensionless variables that encapsulate the geometric and topological features most critical to heat exchange. These parameters form  a set of sub‐unit‐cell descriptors that quantify four complementary attributes of the conduit network: spatial density, connectivity, surface exposure, and geometric uniformity:

- Spatial density (*N*): The number of intrinsic conduits per unit cell (Figure 3a). This quantity is a topological invariant, determined solely by the crystallographic space group symmetry of each TPMS architecture.
- Connectivity (Euler index, χ): A quantitative measure of conduit interconnection complexity. For each TPMS, conduit connectivity is uniquely prescribed by its topology and symmetry, and decreases as |χ| increases (Figure 3b; see Figure 3 caption for details).
- Specific surface area (*S*/*V* ^2/3^): The surface area (*S*) normalized by the volume (*V*) to the two‐thirds power, where the exponent 2/3 provides a scale‐invariant measure that characterizes the surface utilization efficiency of each conduit, independent of its absolute size (Figure 3c). At the unit‐cell level, the corresponding specific surface area scales with the number of conduits as [*S*/*V* ^2/3^]_Unit cell_ = *N* ^1/3^  × [*S*/*V* ^2/3^]_Conduit_ (See Equations S5–S8).
- Uniformity $\left(\bar{d}/\sigma \right)$: The ratio of the mean conduit diameter $\left(\bar{d}/\sigma \right)$ to its standard deviation σ, computed from the Voronoi distance‐to‐boundary field (Figure 3d). Unlike the well‐established volumetric hydraulic diameter—which inherently overlooks cross‐sectional variations [27, 28, 29, 30]—the Voronoi‐based descriptor explicitly captures these spatial fluctuations (Section S2.5). Its effectiveness for characterizing TPMS architectures in heat exchange applications is confirmed and recommended by a recent independent study [31].

These descriptors capture the architecture across scales. The parameters S/V^2/3^ and $\left(\bar{d}/\sigma \right)$ characterize the geometry of individual intrinsic conduits, whereas *N* and |*χ*| encode how these conduits assemble, branch, and interlink across the full TPMS architecture. Although derived from sub‐unit‐cell dissection, the descriptors maintain an intrinsic link to the unit‐cell scale—for instance, *N* directly quantifies the number of intrinsic conduits per unit cell, preserving the design logic of periodic architectures. Collectively, these parameters establish a previously inaccessible, sub‐unit‐cell–resolved design framework that transcends the limitations of unit‐cell averaging while remaining compatible with established manufacturing and modeling conventions.

It should be noted that the wall thickness of real‐world metallic TPMS structures is constrained by manufacturing capability (e.g., a minimum of ∼200 µm for robust metal L‐PBF fabrication). In heat‐exchange applications, TPMS structures typically operate at high porosities (>70%), corresponding to relative densities ρ_*RD*_ < 30%. For a given TPMS architecture, the spatial density *N* is fixed, and conduit uniformity and connectivity remain effectively unchanged as wall thickness varies within this relevant porosity range. Consequently, the influence of wall thickness manifests primarily through its effect on the total surface area. As shown in Table S10, within this practically relevant porosity range, the total surface area of TPMS architectures depends only weakly on wall thickness. Therefore, the effect of wall thickness can be regarded as secondary relative to the dominant geometric roles of conduit spatial density and uniformity.

To assess whether these four variables form a minimal and physically complete basis for TPMS transport within the present theoretical descriptor framework, we re‐indexed an 18‐parameter descriptor pool encompassing nearly all geometric and topological quantities known to influence transport. Using formula‐based reduction relations (Figure S6 and Table S8), we demonstrate that every descriptor—whether geometric or topological, and whether defined at the unit‐cell or sub‐unit‐cell scale—maps deterministically onto one of the four variables (*N*, *S*/*V* ^2/3^, $\left(\bar{d}/\sigma \right)$, |*χ*|) This result shows that the high‐dimensional descriptor space of TPMS architectures collapses onto a compact, non‐redundant, and physically interpretable sub‐unit‐cell–based descriptor set applicable within the present analytical framework, confirming these four variables as the governing parameters for TPMS transport behavior.

## 3 Sub‐Unit‐Cell Profiling of TPMS Transport Efficiency

**FIGURE 4:**
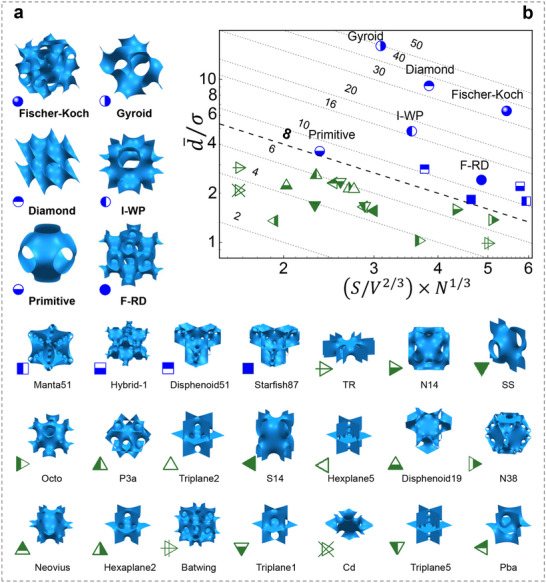
Screening the TPMS design space for high heat‐transfer efficiency using a sub‐unit‐cell framework. (a) Library of 27 representative TPMS architectures was analyzed in this study. (b) Performance map constructed from the combined channel‐uniformity–geometric‐efficiency metric $\left(\bar{d}/\sigma \times \xi \right)$, illustrating the relative heat‐exchange potential of each topology.

Our sub‐unit‐cell framework provides a topological tool for mapping potential TPMS heat‐exchange performance. We apply this framework to 27 commonly reported TPMS topologies (Figure 4a; 1), including benchmark geometries such as the Schwarz surface [33]. To translate sub‐unit‐cell geometry into unit‐cell‐scale transport metrics, we focus on two intrinsic conduit descriptors: (i) $\left(\bar{d}/\sigma \right)$, a channel‐uniformity metric; and (ii) ξ  =  *S*/*V* ^2/3^ × *N* ^1/3^ (see Equations S5–S8 for formulation), representing the available heat‐exchange surface per unit packing density in a TPMS structure. Figure 4b plots $\left(\bar{d}/\sigma \right)$ against ξ for these TPMS topologies. As detailed in Section S2.8, topologies with $\bar{d}/\sigma <5$ exhibit large cross‐sectional fluctuations, ranging from visible necking to geometric fragmentation, and are therefore unsuitable for high‐efficiency heat‐exchange applications. This hydraulic acceptability criterion ($\bar{d}/\sigma >5$) excludes most TPMS designs, leaving Gyroid, Diamond, Fischer–Koch, and I‐WP as the only four viable candidates —all occupying the highest $\bar{d}/\sigma$ band in Figure 4b.

To extend the comparison and provide a broader reference, we also include Primitive (which exhibits the highest $\left(\bar{d}/\sigma \right)$ among the remaining TPMS in Figure 4a) and F‐RD (which shows relatively high values of both $\left(\bar{d}/\sigma \right)$ and ξ). The specific choice among F‐RD, Disphenoid51, Manta51, and Hybrid‐1 (Figure 4b) does not alter the overall conclusion, because (i) none satisfies the $\bar{d}/\sigma >5$ criterion, and (ii) their $\left(\bar{d}/\sigma \times \xi \right)$ values are comparable (≈ 10–12).

The dashed lines in Figure 4b are not intended to impose artificial clustering. Instead, they represent iso‐performance contours of the combined metric $\left(\bar{d}/\sigma \times \xi \right)$, providing a rapid visual guide to expected performance rather than indicating discrete groupings. Higher contours indicate potentially greater performance, particularly when accompanied by a high $\left(\bar{d}/\sigma \right)$ ratio.

To establish a quantitative performance predictor informed by sub‐unit‐cell descriptors, we define a dimensionless performance quotient, *η*, based on classical fluid‐mechanics scaling [34, 35]. This quotient integrates the four intrinsic descriptors into a power‐law expression:

(1) $$\eta ={\left(S/V^{2/3}\right)}^{m}\times {\left(\bar{d}/\sigma \right)}^{n}\times N^{p}\times {\left|\chi \right|}^{-q}$$

It should be emphasized that, within Equation (1), the exponents (*m*, *n*, *p*, *q*) serve solely to parameterize their relative contributions rather than to introduce new physical mechanisms. Conduit uniformity $\left(\bar{d}/\sigma \right)$ quantifies hydraulic regularity, spatial density *N* captures the packing efficiency of the conduit network, surface utilization (*S*/*V* ^2/3^) measures available interfacial area, and topological connectivity |χ| reflects branching richness. The role of the exponents is therefore to modulate the influence of each descriptor within Equation (1), enabling the metric to adapt across different TPMS geometries and transport objectives while preserving clear physical interpretability.

**FIGURE 5:**
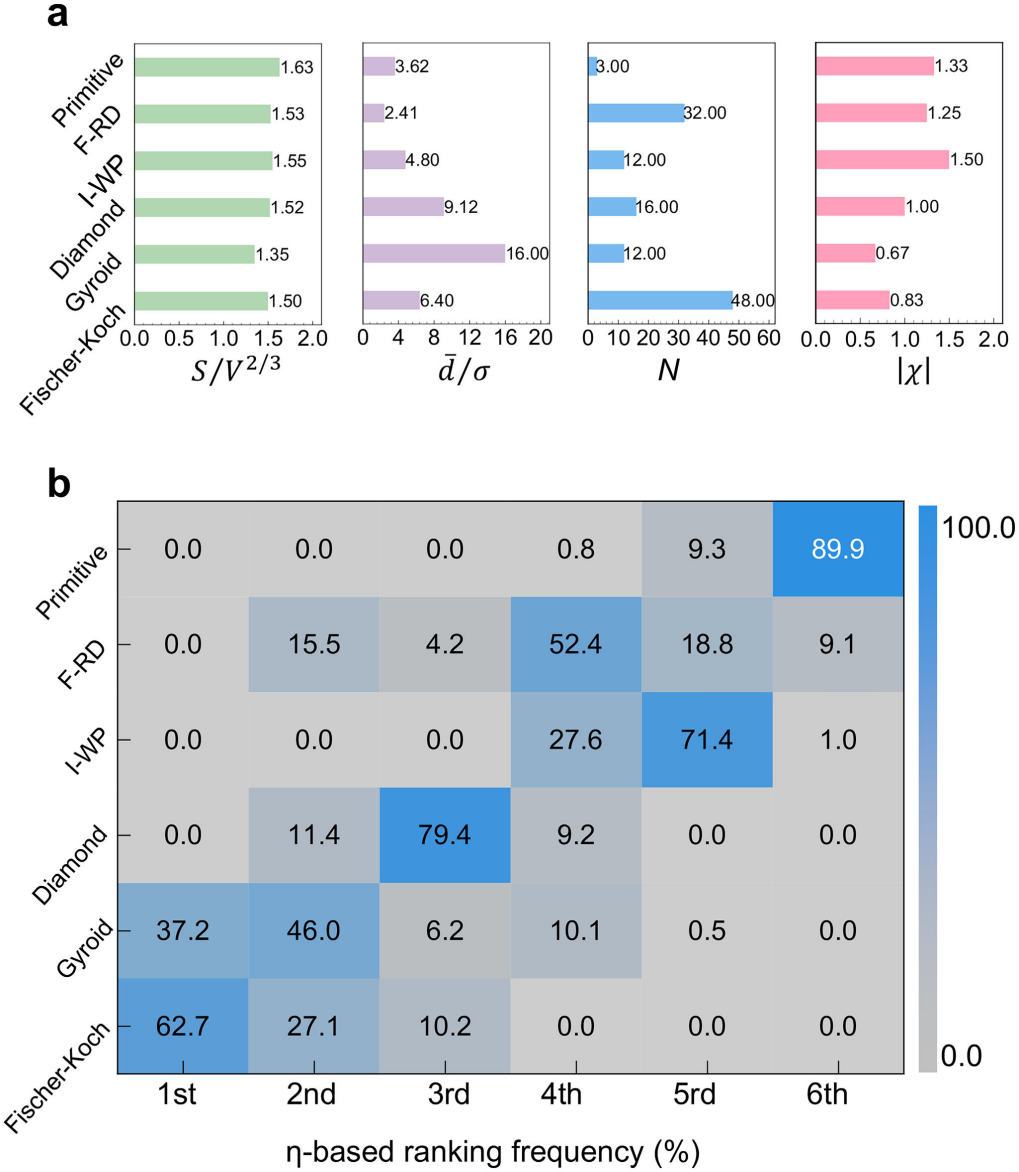
A robust performance hierarchy revealed by global sensitivity analysis of the sub‐unit‐cell performance quotient, *η*. (a) The four fundamental sub‐unit‐cell descriptors—surface utilization (*S*/*V* ^2/3^), geometric uniformity ($\left(\bar{d}/\sigma \right)$), topological complexity (|χ|), and spatial density (*N*)—for the six representative TPMS structures. (b) η‐based frequency map over a wide index space (m, n, p, q) ∈ [0,10] (step = 0.2). For each parameter combination, the six TPMS are ranked by *η* (largest = rank 1, smallest = rank 6); the plot shows the empirical frequencies of these ranks accumulated across all combinations. For further details, see Section S2.10.

We performed a comprehensive parameter sweep across the exponent space, with *m, n, p, q* ∈ [0, 10] at a resolution of 0.2, generating 6.76 million unique combinations. For each combination, the performance quotient *η* was computed for six representative TPMS designs using their intrinsic descriptors (Figure 5a), producing a ranking frequency distribution for each structure. The aggregated results (Figure 5b) reveal a strikingly stable performance hierarchy: Fischer–Koch, Gyroid, and Diamond overwhelmingly dominate the top rankings, with Fischer–Koch consistently occupying first place. I‐WP, F‐RD, and Primitive form a distinct, lower‐performing tier, with Primitive emerging as the least efficient.

To test the robustness of the performance hierarchy, we conducted two additional parameter sweeps: (i) exponents in [0, 10] with a step size of 0.5, and (ii) exponents in [0, 6] with a step size of 0.2, as detailed in Figure S12. The ranking remained stable across both scans, with only minor fluctuations at the boundaries of the exponent space. This global sensitivity analysis confirms that the performance hierarchy is an intrinsic property of the TPMS geometries, not an artifact of specific parameter choices.

**FIGURE 6:**
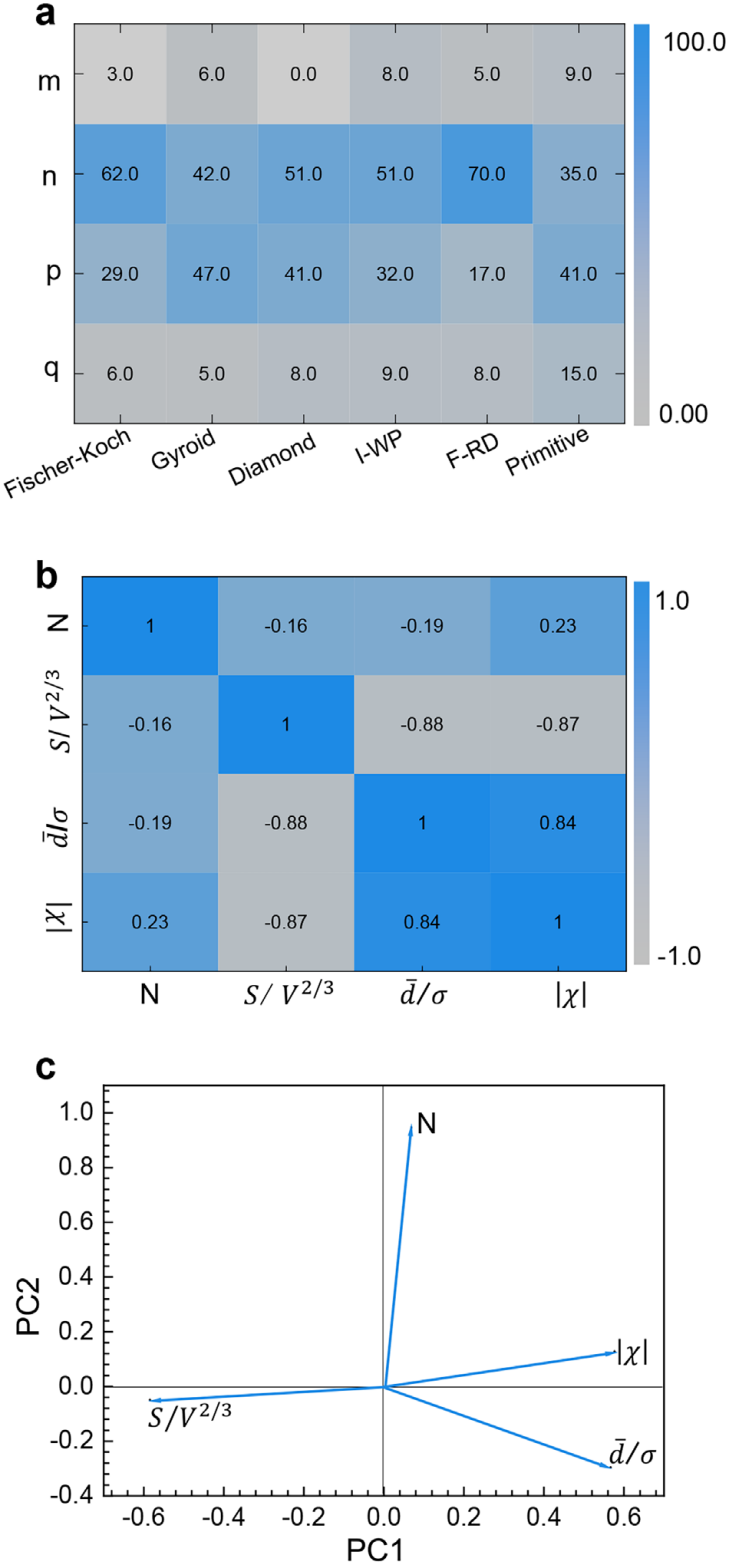
Identifying the dominant geometric drivers of TPMS transport performance. (a) Global sensitivity analysis. The plot shows the first‐order Sobol sensitivity indices for the four descriptors, quantifying their individual contributions to the variance in the performance ranking. Exponents *n* for conduit uniformity ($\left(\bar{d}/\sigma \right)$) and *p* for conduit spatial density (*N*) in Equation (1) dominate the output variance. (b) Correlation matrix. The plot reveals pairwise linear correlations between the four structural descriptors, highlighting the orthogonality of spatial density (*N*) to other parameters. (c) Principal component analysis. The loading plot for the first two principal components (PC1, PC2) shows the contribution and grouping of the four descriptors, confirming that *d̅/σ* and *N* load strongly on separate, orthogonal axes.

To identify the origin of this robustness, we analyzed the global sensitivity indices. Variations in the exponents *n* and *p*—associated with uniformity ($\left(\bar{d}/\sigma \right)$) and spatial density (*N*), respectively—collectively accounted for over 75% of the output variance (Figure 6a). This identifies flow uniformity and conduit packing density as the dominant geometric determinants of TPMS transport performance. Principal component analysis (PCA) and correlation analysis (Figure 6b,c) corroborate this finding: PC1 is dominated by $\left(\bar{d}/\sigma \right)$, while PC2 is primarily controlled by *N*. The weak correlations of *N* with other descriptors (<0.5) underscore its independent role in setting the global routing density.

Building on this mechanistic insight, we condensed Equation (1) into a simplified predictive model by setting *m* = *q* = 0 and *n* = *p* = 1, isolating the dominant terms of uniformity and density:

(2) $$\eta^{\prime }\propto \left(\bar{d}/\sigma \right)\times \left(N\right)$$

The performance scores calculated from Equation (2) are: Fischer–Koch (307.20), Gyroid (192.00), Diamond (145.90), F‐RD (77.10), I‐WP (57.60), Primitive (10.90). Despite its simplicity, this reduced model accurately reproduces the full global sensitivity analysis‐based ranking from Equation (1) and cleanly separates high‐ from low‐efficiency structures.

Thus, by integrating multivariate, sensitivity, and principal component analyses, we have identified geometric uniformity and conduit density as the two dominant structural determinants of TPMS heat‐exchange efficiency. Their dominance arises because these descriptors impose system‐limiting constraints that cannot be offset by surface area or connectivity alone. Non‐uniformity induces flow maldistribution and local bottlenecks, disproportionately raising viscous dissipation; increased spatial density enhances flow redirection and boundary‐layer renewal. Surface utilization and topological connectivity describe geometric capacity whose benefits require well‐distributed, dense flow. Consequently, sensitivity analysis reveals a physically filtered hierarchy in which uniformity and density are primary enablers, while surface area and connectivity are secondary modifiers. This sub‐unit‐cell decoding, combined with a data‐driven ranking framework, establishes a predictive and mechanistic design paradigm for TPMS systems. It enables the rapid identification of high‐performance TPMS architectures directly from their geometric descriptors, bypassing the need for costly simulations or experiments.

Benchmarking against conventional TPMS analyses (Table S2) shows that prior studies rely almost exclusively on unit‐cell–averaged metrics such as porosity and surface area. By contrast, our framework is formulated at the sub‐unit‐cell level, which represents a more fundamental and intrinsic building block of TPMS architectures. Rather than isolating individual geometric parameters through idealized control‐variable experiments, we seek to capture how intrinsic structural features collectively govern transport behavior in both statistical and physical–mechanistic senses.

## 4 Validation of the Sub‐Unit‐Cell Framework for Heat‐Exchange

We validated the sub‐unit‐cell framework and its predictive models (Equations (1), (2)) using heat exchange as a test case. This choice is motivated by escalating thermal demands of artificial intelligence (AI) systems^2^[36] and the growing recognition of TPMS architectures as next‐generation thermal metamaterials.t

**FIGURE 7:**
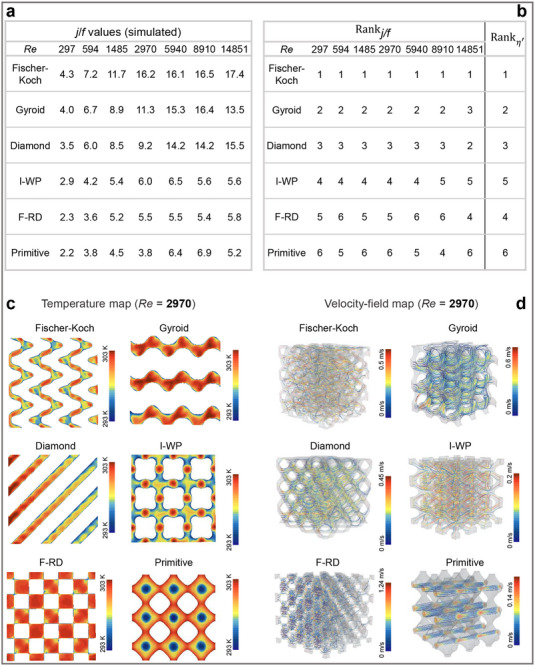
Simulation‐based assessment of liquid‐cooling performance in six selected TPMS heat exchangers (assuming fabricated in copper). (a) Simulated *j*/*f* for all six TPMS architectures over Reynolds numbers spanning laminar (*Re*: 297, 594), transitional (*Re*: 1485, 2970), and fully turbulent (*Re*: 5941, 8911, 14851) regimes. The highlighted *Re* = 2970 is used as the reference condition in Figure 9. (b) *j/f*‐based rankings (Rank*_j/f_*) of six TPMS architectures at seven Reynolds numbers, compared with the Re‐independent *η′‐*predicted ranking (Rank*_η′_*, rightmost column), where smaller rank values indicate better performance (1 = 1st, 6 = 6th). (c) Cross‐sectional temperature distributions at *Re* = 2970, revealing distinct thermal‐gradient patterns, with Fischer–Koch, Gyroid, and Diamond exhibiting more uniform cooling. (d) Velocity‐field maps at *Re* = 2970, illustrating topology‐dependent flow organization and correlating with conduit uniformity ($\left(\bar{d}/\sigma \right)$).

To assess the robustness of the predicted performance hierarchy, we conducted high‐fidelity simulations of six representative copper TPMS structures under liquid‐cooling conditions, spanning Reynolds numbers (*Re*) from 297 to 14 851 (Figure 7a–b). Figure 7a shows the simulated *j/f* values across the investigated *Re* range, while Figure 7b compares the corresponding *j/f*‐based rankings with those predicted using the reduced geometric descriptor *η′*. Agreement between predicted and simulated rankings was quantified using Spearman's rank correlation coefficient (*ρ*, where *ρ* = 1 denotes perfect agreement; see Sections S2.10–S11). The resulting *ρ* values are 1.0, 0.9, 1.0, 1.0, 0.8, 0.8, and 0.9 for *Re* = 297–14851, indicating strong monotonic agreement and preservation of the performance hierarchy across flow regimes.

As comparators to Equation (2), alternative formulations involving *S*/*V* ^2/3^ (Equations S9–S12) were evaluated and found to exhibit markedly poorer agreement with the simulated *j*/*f* rankings, confirming that conduit uniformity ($\left(\bar{d}/\sigma \right)$) and channel multiplicity *N* constitute the minimal and robust descriptors governing performance ranking. Building on this ranking‐level validation, a supplementary regression analysis shows that the sub‐unit‐cell descriptors can be combined into a quantitative scaling relation to predict *j/f* across the investigated Reynolds‐number range (Equations S13–S14).

Building on the demonstrated cross‐*Re* robustness, we return to Figure 7a and focus on *Re* = 2970 as a representative operating condition, as it lies within the stable turbulent regime commonly adopted for compact heat exchangers. At this Reynolds number—corresponding to the highlighted column in Figure 7a—a clear performance hierarchy emerges: Fischer–Koch (16.2), Gyroid (11.3), and Diamond (9.2) consistently outperform I‐WP (6.0), F‐RD (5.5), and Primitive (3.8). The corresponding temperature fields and velocity distributions at *Re* = 2970 are shown in Figure 7c–d, revealing distinct flow organization and thermal transport pathways associated with each topology.

The geometric origins of this hierarchy are directly interpretable within our framework. Fischer–Koch combines high conduit uniformity ($\left(\bar{d}/\sigma =6.40\right)$) with the highest spatial density (*N* = 48), yielding the strongest overall performance. Gyroid exhibits exceptional uniformity (16.00) but a substantially lower spatial density (12), accounting for its second‐place ranking. In contrast, F‐RD offers only a moderate spatial density (32) with poor uniformity (2.41), while Primitive—with only three intrinsic conduits— is fundamentally constrained by limited pathway availability. Collectively, these results substantiate the central insight of our analysis: thermal efficiency in TPMS structures is governed by the combined effects of conduit uniformity, which regulates flow organization, and conduit spatial density, which determines both the multiplicity of flow pathways and the extent of available heat‐transfer surface.

**FIGURE 8:**
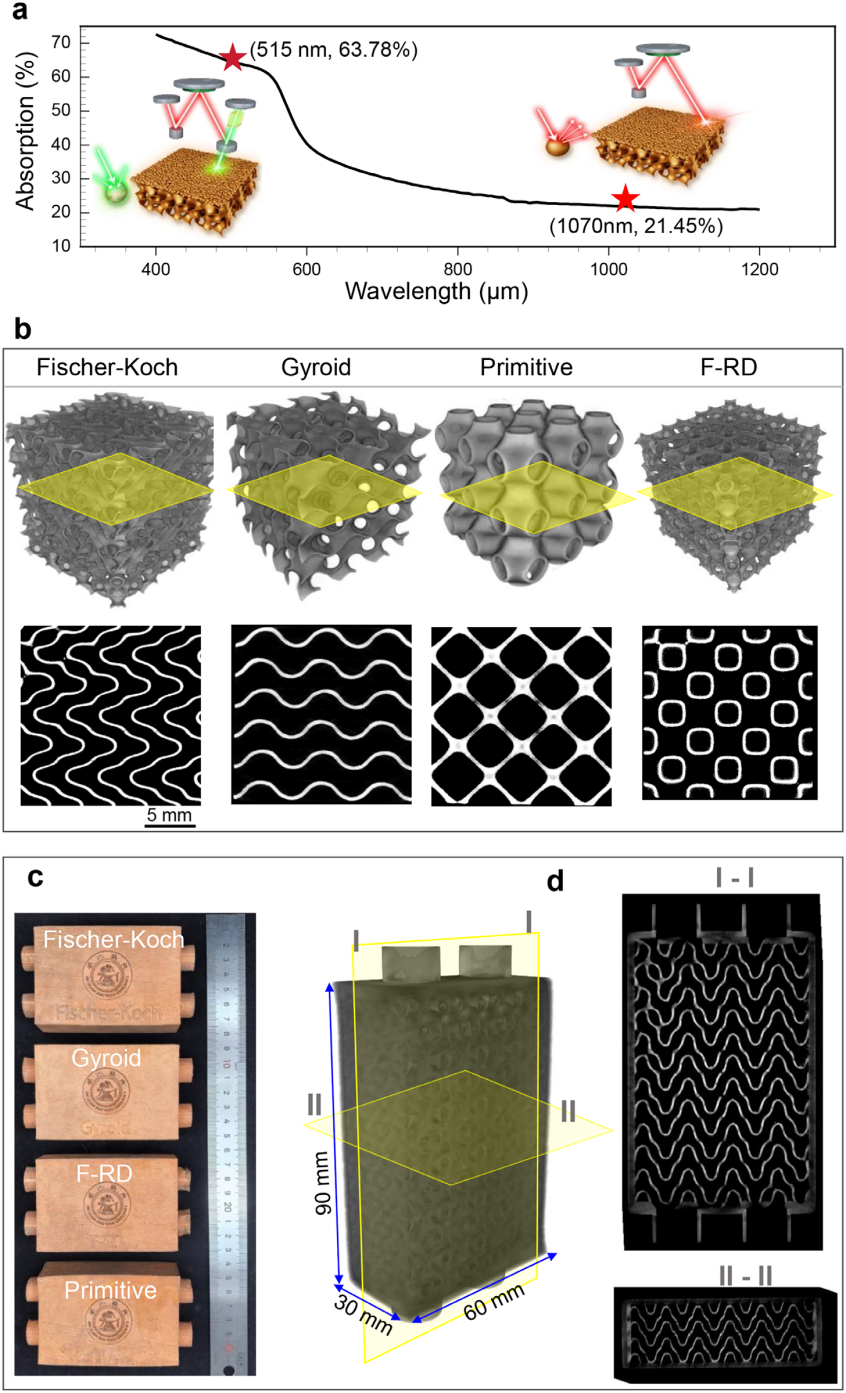
Fabrication of high‐quality copper TPMS architectures via green‐laser powder bed fusion (GL‐PBF). (a) Enhanced energy absorption of pure copper powder under green laser (515 nm) compared to a conventional infrared laser (1070 nm), which enables robust processing ^3^. (b) μ‐CT reconstructions and representative cross‐sectional slices of four open TPMS architectures, demonstrating uniform strut formation, continuous channel networks, and consistently low defect content (<0.3 vol.%). These results confirm that the selected GL‐PBF parameters ensure geometrically faithful and repeatable fabrication across distinct TPMS topologies. (c) Photographs of fully enclosed TPMS heat exchangers fabricated using the same process parameters, illustrating the successful transition from open architectures to device‐level, encapsulated cooling components. (d) Cross‐sectional view of an enclosed Fischer–Koch heat exchanger, revealing that the internal TPMS walls remain uniform and fully dense after encapsulation, confirming robust internal quality and manufacturability for practical thermal‐management applications. For further design and manufacturing details, see Section S3.

To transition from simulation to physical validation, we established the fabrication readiness of high‐thermal‐conductivity copper TPMS metamaterials using a GL‐PBF process. Copper's high reflectivity to conventional infrared lasers makes it notoriously difficult to process, but green‐laser irradiation significantly improves absorptivity 3, thereby enabling robust printability (Figure 8a). Using this approach, we first fabricated open TPMS architectures (Figure 8b). µ‐CT reconstructions confirm excellent geometric fidelity, uniform wall thickness, continuous channels, and very low defect levels (<0.3 vol.%), demonstrating reliable and topology‐independent manufacturability.

Building on this baseline, we fabricated fully encapsulated copper TPMS heat exchangers under identical conditions (Figure 8c–d). The encapsulated design imposes stricter requirements on wall density, shell integrity, and channel isolation. High‐resolution X‐ray computed tomography (CT) of a copper Fischer–Koch device (Figure 8d) confirms uniform, dense internal walls after encapsulation. Sealing quality and flow‐channel isolation were further verified through flow visualization and leakage tests (Video S1).

**FIGURE 9:**
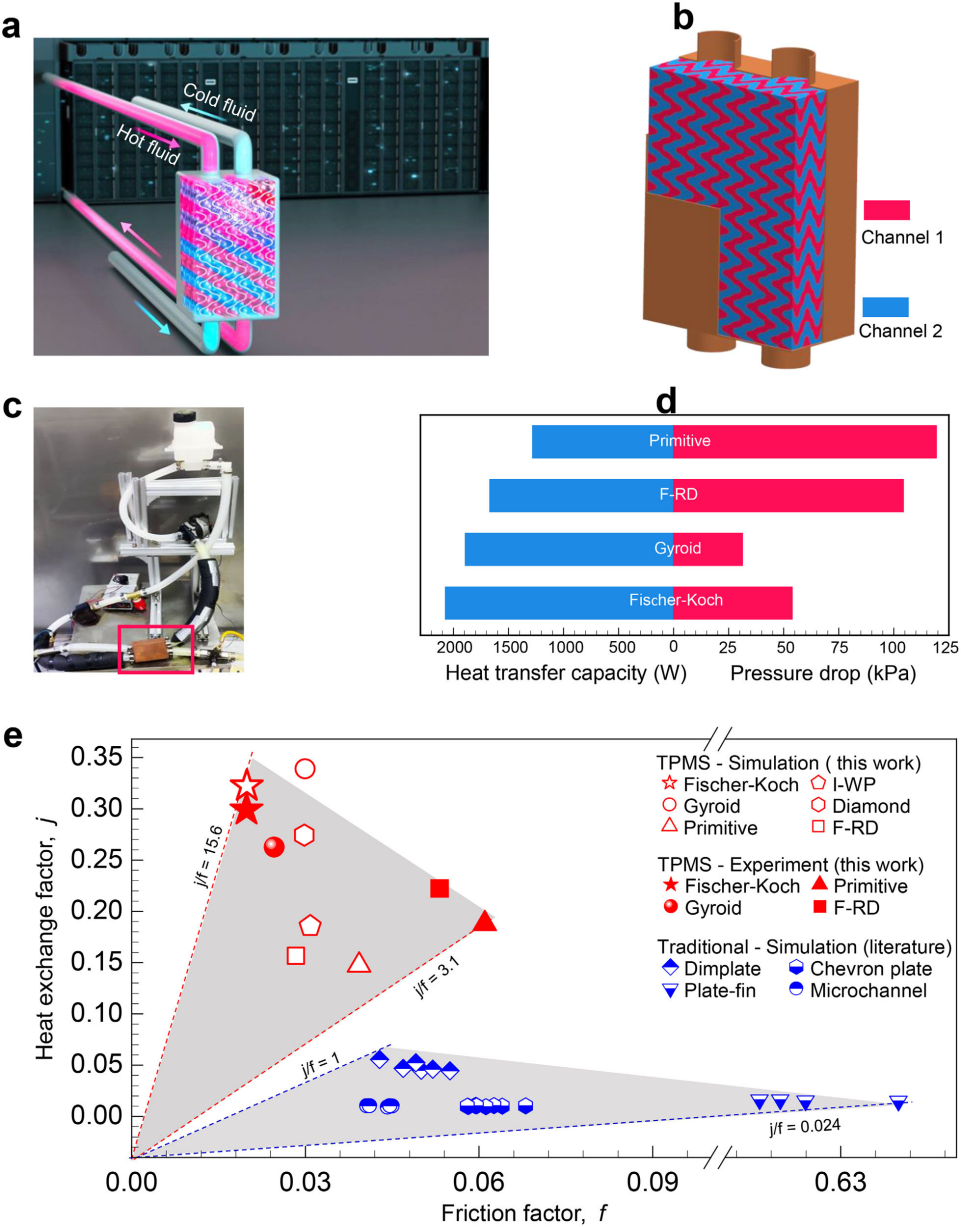
Experimental validation of copper TPMS heat exchangers demonstrates a paradigm shift in liquid‐cooling efficiency. (a) Schematic integration of a TPMS heat exchanger within an AI data‐center cooling loop. (b) CAD model of a dual‐channel TPMS heat exchanger, illustrating two interpenetrating yet fully isolated flow networks within a Fischer‐Koch architecture. (c) Custom test rig for liquid‐cooling performance, with the unit under test highlighted (red box). (d) Experimentally measured heat transfer and corresponding pressure drop for the four TPMS architectures under the same liquid‐cooling condition as Figure 7a–d (*Re* = 2970). (e) Performance breakthrough. The measured *j/f* efficiency metric for the TPMS heat exchangers shows a 150‐fold enhancement over the performance range of traditional heat exchangers (Table S1), validating the predictive power of the sub‐unit‐cell design framework. For more details, see Section S4.

With process fidelity established, we evaluated the liquid‐cooling performance of the fabricated copper TPMS heat exchangers under conditions representative of data‐center thermal management (Figure 9a). Four designs spanning the simulated performance range—Fischer–Koch, Gyroid, F‐RD, and Primitive—were experimentally characterized under the same representative flow condition (Re ≈ 2970) used in the simulations (Figure 9b). A custom test rig (Figure 9c) enabled simultaneous measurement of heat‐transfer capacity and pressure drop (Figure 9d) and *j/f* ratios (Figure 9e).

The simulations predict real‐world performance, capturing both the performance ranking and the correct order of magnitude. Specifically, for the Fischer–Koch, Gyroid, Primitive, and F‐RD architectures, simulated j/f values are 16.2, 11.3, 5.5, and 3.8, compared with measured values of 15.6, 10.7, 4.2, and 3.1, respectively. Notably, whereas conventional heat exchangers typically achieve j/f values of 0.024–1 (mostly <0.1), the Fischer–Koch TPMS architecture achieves a j/f of 15.6, corresponding to a 156‐fold enhancement. This exceptional performance is an intrinsic property of the Fischer–Koch architecture itself. The sub‐unit‐cell framework identified this architecture a priori as a high‐potential candidate, and the predicted superiority was subsequently confirmed through experiments. While this framework also provides a rational basis for the future design of high‐ j/f TPMS architectures, the present study focuses on demonstrating its predictive capability and validation.

Collectively, our computational, manufacturing, and experimental results confirm that transport efficiency in TPMS architectures is governed by sub‐unit‐cell geometry, rather than by traditional unit‐cell descriptors. The predictive power of the minimal two‐parameter model—based solely on conduit uniformity ($\left(\bar{d}/\sigma \right)$) and spatial density (*N*)—for full‐scale thermal performance underscores the novelty of our approach. This framework distills complex architectures into fundamental geometric drivers, establishing a rational, data‐driven paradigm for the design of highly efficient TPMS thermal systems.

Although heat exchange is used here as a representative validation case, the proposed sub‐unit‐cell descriptors and performance quotient are not restricted to thermal transport. The framework is formulated in terms of geometric regularity, pathway multiplicity, interfacial availability, and network topology—features common to a broad range of transport processes. For mass transport and reactive flow systems, these descriptors govern permeability, residence‐time distribution, and effective surface utilization, while in electrochemical systems, they relate to ionic pathway redundancy, tortuosity, and accessible active area. In such cases, the same geometric descriptor set can be retained, with the response metric (e.g., *j*/*f*) replaced by an appropriate mass‐ or charge‐transport figure of merit.

## 5 Summary

We introduce a design paradigm that decodes TPMS architectures into their fundamental sub‐unit‐cell transport conduits. We show that each TPMS can be resolved into a network of identical intrinsic conduits oriented in different directions, whose geometry and connectivity are uniquely determined by topology. This intrinsic sub‐unit‐cell conduit network directly governs flow and transport behavior. By replacing conventional unit‐cell‐averaged descriptions with this sub‐unit‐cell perspective, we reveal that TPMS thermofluidic performance is governed primarily by two factors: conduit uniformity and conduit spatial density. This sub‐unit‐cell logic establishes a physics‐based framework for evaluating TPMS architectures.

From this foundation, we derive predictive descriptors that directly link local conduit geometry to system‐level performance. The approach is validated through five independent and convergent lines of evidence: (1) a sensitivity‐driven ranking model, (2) quantitative regression capturing the majority of performance variance, (3) high‐fidelity CFD simulations reproducing the predicted hierarchy, (4) experimental measurements using additively manufactured copper TPMS heat exchangers confirming the ranking and demonstrating up to a 156‐fold enhancement in j/f performance, and (5) theoretical analysis showing that the descriptor set forms a minimal, non‐redundant geometric basis.

These results demonstrate that the sub‐unit‐cell conduit framework provides a mechanistically grounded and predictive tool for screening and ranking TPMS architectures, exemplified by the successful identification and experimental validation of the high‐performance Fischer–Koch structure. This work shifts TPMS design from descriptive classification to performance‐guided exploration, offering a scalable and generalizable strategy for next‐generation architected transport materials.

## 6 Materials and Methods

### 6.1 TPMS Design and Geometric Analysis

TPMS architectures were generated through two complementary approaches. A subset was created mathematically using explicit equations (Table S3), where the parameter *t* controlled the surface offset. The solid lattice region between two offset surfaces was defined via a Boolean intersection with a bounding cuboid, performed using K3DSurf (a free software listed under Presentation Tools in Audio & Multimedia) and Siemens NX (NX Nastran, Siemens Industry Software Inc., USA).

For the remaining TPMS architectures, we complemented the equation‐based generation with a surface‐energy–minimization workflow using Surface Evolver (v2.70). Custom Python scripts (v3.11.1) were developed to compute structural diameters, while Mathematica (v13.3, Wolfram Research) was used for Voronoi‐based visualization and extraction of intrinsic transport domains. VESTA (v3.5.8), in conjunction with the RCSR database, was employed to derive the TPMS skeletal networks, which were subsequently rendered in Siemens NX (v12). Global sensitivity analysis (GSA) of the resulting descriptor space was performed using MATLAB (R2022b, MathWorks).

### 6.2 Green Laser‐Based Powder Bed Fusion Manufacturing

Green‐laser powder bed fusion (GL‐PBF) was carried out using the TruPrinting 1000 Green Edition system (TRUMPF Laser‐ und Systemtechnik GmbH). Argon gas‐atomized copper powder (SHANGHAI ST‐NANO SCIENCE AND TECHNOLOGY CO., LTD) served as the feedstock, and a Ti‐6Al‐4V build plate was employed with a preheating temperature of 100 °C. Following fabrication, the samples were allowed to cool inside the machine to room temperature (25 °C) before being removed from the substrate via wire cutting. The process parameters were: laser power 275 W, scanning speed 1000 mm/s, hatch spacing 80 µm, and layer thickness 30 µm.

### 6.3 Characterization

**SEM**: Copper powder morphology was examined using a TESCAN LYRA3 scanning electron microscope (SEM) operated in secondary‐electron mode at an accelerating voltage of 20.0 kV.

**CT**: X‐ray computed tomography (XT H 450, Nikon Metrology Inc.) was used to characterize the TPMS heat exchangers (Figure 8c,d; Figure S15). The imaging parameters were: FDD = 1019 mm, tube current = 565 µA, tube voltage = 435 kV, and an effective pixel size of 67.9 µm. Image reconstruction was performed using Dragonfly (Object Research Systems Inc.) to assess morphology and internal defects. CT scans of solid copper reference samples were also conducted on the XT H 450, using a tube voltage of 220 kV, tube current of 500 µA, and a pixel size of 25.6 µm.

**Spectrophotometer**: The optical absorption of copper powder was measured using a UV–vis‐NIR spectrophotometer (Lama D950). The wavelength range was 175–3300 nm, with UV/VIS spectral bandwidths of 0.01–5 nm and NIR bandwidths of 0.2–20 nm.

### 6.4 Simulation of Liquid Cooling Performance

6.4.1

Mathematical Model: The flow and heat‐exchange behavior of each TPMS architecture was evaluated using CFD simulations implemented in Simcenter STAR‐CCM+ 2021. The conjugate heat transfer, single‐phase flow module was employed to simultaneously resolve heat conduction within the solid and convective transport within the fluid. An incompressible Navier–Stokes fluid model with constant density and viscosity, together with the RNG *k–ε* turbulence formulation, was employed to resolve the flow through TPMS channels [37].

6.4.2

Geometry and Meshing: To enable direct comparison across architectures, a 3 × 3 × 3 array of TPMS unit cells was used for all simulations. The computational domain consisted of a solid copper region and two independent fluid regions (hot‐side and cold‐side channels), illustrated in Figure S16 using the Gyroid as an example. The relative density of all TPMS structures was fixed at 38%, which primarily determined the thickness of the solid web without significantly altering the fluid domain.

Unstructured meshes were generated for the fluid regions. Mesh‐independence was assessed by monitoring inlet–outlet pressure drop at varying mesh densities (Table S13). The pressure difference decreased with decreasing minimum node size and stabilized once the minimum size fell below 0.0023 mm, corresponding to more than 159,825 total elements. This value was therefore selected for all subsequent simulations, ensuring that the results were mesh‐independent.

6.4.3

CFD Setup and Boundary Conditions: Six TPMS architectures—Primitive, I‐WP, Fischer–Koch, Diamond, Gyroid, and F‐RD—were simulated under identical boundary and geometric conditions: unit‐cell size of 3 mm and relative density of 38%. Copper was assigned to the solid domain, and water was used as the working fluid in both channels, with material properties obtained from the STAR‐CCM+ built‐in library.

Flow directions for the hot and cold fluids are shown in Figure S16. Input flow rates were selected based on measured values from conventional heat exchangers, and equal volumetric flow rates were assumed for the hot and cold channels. The specific inlet velocities and temperatures for both fluids are listed in Table S16. Outlet boundaries were set as pressure outlets, and all remaining walls were treated as adiabatic. Under these conditions, the simulations yielded the outlet temperature and pressure for each channel. Each CFD run required approximately 5 h of computational time.

### 6.5 Experimental Tests on Liquid‐Cooling Heat Exchangers

The liquid‐cooling performance of the four GL‐PBF–fabricated TPMS heat exchangers was experimentally evaluated using a dedicated test platform. Temperatures at the inlet and outlet of each exchanger were measured using T‐type thermocouples with an accuracy of ±0.1 °C. Water pressure was recorded using an absolute pressure sensor (accuracy ±0.12% of full scale), and volumetric flow rate was monitored with an electromagnetic flowmeter (accuracy ±0.15% of full scale). The test system consisted of two independent liquid circulation loops, with all components listed in Tables S15–S16. A full uncertainty analysis is provided in Table S17.

Prior to testing, the tightness and reliability of the entire fluid circuit were verified, and all thermocouples, pressure sensors, and flowmeters were calibrated. During operation, the pump power on the water side and the inlet conditions on the ethylene‐glycol side were adjusted to reach the prescribed working conditions. The system required approximately 30 min to reach thermal steady state before data acquisition was initiated.

Measurements were logged every 5 s. Once inlet and outlet temperatures, pressures, and flow rates stabilized, data were recorded at 10‐min intervals, and the average of three measurements was used. The inlet temperature on the water side was maintained at ambient temperature with a constant flow rate of 2.5 L/min. On the ethylene‐glycol side, the inlet temperature was set to either 35°C, with a flow rate 10 L/min, as detailed in Table S16. Table S18 details the inlet/outlet temperatures for all TPMS heat exchangers. For example, in the Fischer‐Koch heat exchanger, the cold stream (2.5 L min^−^^1^) heated from 16.4°C to 28.2°C, corresponding to a heat‐transfer rate of 2073.0 W, and the hot stream (10 L min^−^^1^) cooled from 35.2°C to 31.6°C, releasing 2155.9 W. The close agreement between these values confirms well‐balanced heat exchange. The Reynolds numbers (*Re*) were calculated using Equation (3).

(3) $$Re=\frac{\rho uD}{\mu }\;$$

where *ρ* is the liquid density, *u* is the superficial (bulk) velocity defined by the imposed volumetric flow rate divided by the nominal inlet cross‐sectional area of the TPMS core, *μ* is the dynamic viscosity of the liquid, and *D* is the mean conduit diameter obtained from the Voronoi distance‐to‐boundary field (mean of local maximum‐inscribed‐sphere diameters) within the flow domain.

## Author Contributions

H.Z., J.G., and M.Q. performed in conceptualization. H.Z., Y.H., Q.T., Z.N., W.Y., Z.C., C.Q., J.W., J.S., J.C., G.E.S.‐T., and M.Q. performed in Methodology. B.Y., C.Q., C.L., G.E.S.‐T., and R.D. performed in investigation. H.Z. performed in design, fabrication, and experimental characterization. H.Z., J.G., and M.Q. performed in project administration. J.G., B.Y., J.C., J.L., and M.Q. performed in supervision. H.Z. and M.Q. performed in critical analysis. H.Z. and M.Q. performed in writing.

## Conflicts of Interest

The authors declare no conflicts of interest.

## Supporting information




**Supporting File**: advs74663‐sup‐0001‐SuppMat.docx.

**Supporting File**: advs74663‐sup‐0002‐VideoS1.mp4.

## Data Availability

The data that support the findings of this study are available in the supplementary material of this article.

## Notes

1 While topology optimization methods can generate additional TPMS designs, such as those reported in https://doi.org/10.1016/j.addma.2023.103779, these structures often suffer from excessive necking, characterized by low d̅/σ ratios, which compromises their effectiveness for heat exchange applications.

2 According to the IEA's 2022 report (Global data center electricity use to double by 2026 ‐ IEA report), data center electricity use hit 460 TWh in 2022 and, growing 21% annually, may reach 100,000 TWh by 2050.

3 According to the IEA's 2022 report (Global data center electricity use to double by 2026 ‐ IEA report), data center electricity use hit 460 TWh in 2022 and, growing 21% annually, may reach 100,000 TWh by 2050.
