# Supplementary material for: Sub‐Unit‐Cell Logic Governs Transport in TPMS Architectures
Source: Adv Sci (Weinh). 2026 Mar 9;13(29):e23188. doi: 10.1002/advs.202523188 (PMC13205735; doi:10.1002/advs.202523188)
Supplement: Supplementary file 1 — Supporting File: advs74663‐sup‐0001‐SuppMat.docx. [file ADVS-13-e23188-s002.docx]

**Supplementary Material**

# Transport architectures from conventional design to TPMS metamaterials: a review

Transport metamaterials are a class of architected structures engineered to facilitate and control the movement of mass, heat, or momentum in a prescribed manner. These include systems optimized for mass transfer (e.g., diffusion or filtration), heat transfer (e.g., conduction or convection), and fluid momentum transport (e.g., flow distribution or pressure regulation). Among them, Triply Periodic Minimal Surface (TPMS) metamaterials have emerged as a promising platform for such functions due to their continuous, interconnected, and geometrically efficient architectures. In this review, we focus specifically on their role in thermal transport, highlighting key design strategies, reported performance metrics such as the j/f ratio, and the evolution of TPMS-based heat exchangers.

## 1.1 Revisiting the heat–flow trade-off in conventional transport designs

In engineering applications, it is essential to balance heat exchange efficiency and flow resistance. To evaluate this, Focke ^[1]^ introduced the dimensionless factor j/f, where j is the heat exchange coefficient and f represents the friction factor. A higher j indicates better heat transfer, while a higher f reflects greater pressure drop.

The j/f ratio, often called the “area quality factor”, provides a comprehensive measure of heat exchanger performance. Using this metric, we assessed the thermal and flow characteristics of various TPMS architectures, comparing them to conventional heat exchangers. The dimensionless heat exchange factor j for convection is defined in **S-Eq. (1)**.

| $j=\frac{N_{u}}{R_{e}{Pr}^{1/3}}=\frac{\alpha}{\rho\mu c_{p}}{Pr}^{2/3}$ | **(1)** |
| --- | --- |

where Pr is the Prandtl number of the fluid (Pr = 7 for water and Pr = 0.7 for air ^[2]^); $\alpha$ is the convective heat transfer coefficient; $\rho$ is the density of the fluid; $\mu$ is the viscosity of the fluid; $c_{p}$ is the specific heat of the fluid.

The friction factor f was defined as the ratio of the shear stress of the fluid flowing through the unit heat exchange area to the flow kinetic energy of the unit volume as shown in **S-Eq. (2)**.

| $f=\frac{\tau_{w}}{\rho\frac{u^{2}}{2}}=\frac{\frac{\Delta pD_{e}}{4L}}{\rho\frac{u^{2}}{2}}=\frac{\Delta pD_{e}}{2\rho u^{2}L}$ | **(2)** |
| --- | --- |

where $\Delta p$ is the differential pressure of the fluid at the inlet and outlet; $D_{e}$ is the average orifice diameter of the flow channel; $\rho$ is the density of the fluid; $u$ is the velocity of the fluid; L is the length of heat exchange channel. Furthermore, we provide a detailed clarification of the non-dimensionalisation used in the calculation of the j/f factors, as outlined below.

**i) Reference velocity**

The characteristic velocity was defined as the superficial (bulk) velocity, calculated as the imposed volumetric flow rate divided by the total inlet cross-sectional area of the TPMS core (the porosity was identical for all TPMS structures studied in this work). This definition is used throughout to provide a direct, architecture-to-architecture comparison under identical imposed flow rates ^[3]^.

**ii) Characteristic length**

The characteristic length was taken as the mean conduit diameter extracted from the Voronoi distance-to-boundary field, i.e. the mean of the local maximum-inscribed-sphere diameters within the flow domain. This definition provides an intrinsic, direction-independent measure of the effective hydraulic clearance within the labyrinthine TPMS channels and is therefore suitable for comparing complex 3D conduit geometries.

**iii) Pressure-drop definition and friction factor**

The measured pressure drop corresponds to the system-level pressure drop between the upstream and downstream plenums (manifolds) of the encapsulated heat exchanger, i.e. between pressure taps located immediately before the inlet plenum and immediately after the outlet plenum. Consequently, pressure-drop includes both (i) the pressure loss through the TPMS core and (ii) minor losses associated with flow contraction/expansion and distribution within the inlet/outlet plenums. The corresponding friction factor reported in this work is therefore a component-level friction factor ^[4]^.

We emphasize that this definition is appropriate for the present study because all heat exchangers share the same overall encapsulation design and were tested under identical boundary conditions; thus, the manifold-related losses are comparable across samples, and the observed differences in f_overall_ primarily reflect architecture-dependent flow resistance.

**iv) Overall j factor and scope of applicability**

The heat transfer rate was obtained from energy balances on both hot and cold streams. The overall heat transfer coefficient was determined from Q=UAΔT, where A is the heat-transfer area of the TPMS core and ΔT is the log-mean temperature difference. Accordingly, the reported j factor was defined using an overall Stanton number, j_overall_ =St_overall_Pr^2/3^, and thus serves as an overall thermo-hydraulic performance index rather than a strict film-side Colburn j factor ^[5]^. This overall-based comparison is justified here because all TPMS heat exchangers were tested with the same fluids, inlet temperatures, and fixed flow rates on both sides, and the encapsulation design and wall material are consistent; therefore, variations in j_overall_ primarily reflect differences in the TPMS core transport behaviour.

j/f is commonly used to evaluate the thermal performance of heat exchangers. **S-Table 1** collects the reported j/f.

**S-Table 1 Literature values of j/f ratio.**

| Type | Factor j | Factor f | j/f | Experiment or simulation | Literature |
| --- | --- | --- | --- | --- | --- |
| Dimple plate | 0.045 | 0.050 | 0.900 | Experiment | ^[6]^ |
|  | 0.046 | 0.052 | 0.885 | Experiment | ^[7, 8]^ |
|  | 0.047 | 0.047 | 0.998 | Experiment | ^[9]^ |
|  | 0.045 | 0.055 | 0.811 | Experiment | ^[10, 11]^ |
|  | 0.049 | 0.052 | 0.936 | Simulation | ^[12]^ |
|  | 0.043 | 0.056 | 0.771 | Experiment | ^[13]^ |
| Chevron plate | 0.010 | 0.064 | 0.156 | Experiment | ^[14]^ |
|  | 0.010 | 0.063 | 0.167 | Simulation | ^[15]^ |
|  | 0.011 | 0.063 | 0.166 | Experiment | ^[16]^ |
|  | 0.011 | 0.059 | 0.182 | Experiment | ^[17]^ |
|  | 0.009 | 0.061 | 0.155 | Simulation | ^[18]^ |
|  | 0.011 | 0.059 | 0.179 | Experiment | ^[19]^ |
|  | 0.010 | 0.058 | 0.168 | Experiment | ^[20]^ |
|  | 0.010 | 0.068 | 0.149 | Experiment | ^[21]^ |
|  | 0.010 | 0.060 | 0.172 | Simulation | ^[22]^ |
|  | 0.010 | 0.058 | 0.173 | Simulation | ^[23, 24]^ |
| Plate-fin | 0.015 | 0.640 | 0.023 | Experiment | ^[25]^ |
|  | 0.016 | 0.620 | 0.026 | Experiment | ^[26]^ |
|  | 0.015 | 0.624 | 0.024 | Simulation | ^[27]^ |
|  | 0.015 | 0.588 | 0.025 | Experiment | ^[28, 29]^ |
|  | 0.016 | 0.616 | 0.026 | Simulation | ^[30]^ |
| Microchannel | 0.010 | 0.045 | 0.222 | Experiment | ^[31]^ |
|  | 0.010 | 0.041 | 0.255 | Simulation | ^[32]^ |
|  | 0.009 | 0.044 | 0.205 | Experiment | ^[33]^ |
|  | 0.010 | 0.045 | 0.230 | Experiment | ^[34]^ |
|  | 0.010 | 0.041 | 0.251 | Simulation | ^[35]^ |

## 1.2 Rethinking TPMS heat exchangers: limits of the unit-cell perspective

The thermal performance of TPMS structures is summarized in **S-Table 1**. Reviews by Kirttayoth Yeranee et al. ^[36]^ and Krzysztof Dutkowski et al. ^[37]^ have explored TPMS-based heat exchangers. Most studies on TPMS structures as heat exchangers have been conducted through simulations, with very limited experimental research. Notably, there are only two papers that investigates the thermal performance of copper TPMS metamaterials. Furthermore, there is a notable lack of comprehensive analysis linking thermal performance to topological parameters, as highlighted in **S-Table 2**. As a result, the relationship between TPMS topology and thermal performance remains inadequately established, with current research primarily relying on a trial-and-error approach in selecting TPMS structures.

**S-Table 2 Summary of reported studies on the thermal performance of TPMS.**

| **Single/Two fluid domain** | **Fluid** | **Experiment or/and simulation** | **Constituent**  **material** | **Structure type** | **Unit-cell-level** | **References** |
| --- | --- | --- | --- | --- | --- | --- |
| Single | Air | Simulation | / | Gyroid, Diamond | **√** | ^[38]^ |
| Single | Air | Simulation | / | Gyroid, Diamond | **√** | ^[39]^ |
| Two | CO_2_ | Simulation | / | Gyroid, Diamond | **√** | ^[40]^ |
| Single | Air and metal | Simulation | / | Kelvin, Gyroid, I-WP, Primitive | **√** | ^[41]^ |
| Single | Air | Simulation | / | Gyroid, Diamond | **√** | ^[42]^ |
| Single | Air | Simulation | / | Diamond | **√** | ^[43]^ |
| Single | Air | Simulation | / | Gyroid, Diamond | **√** | ^[44]^ |
| Single | Air | Simulation | / | Diamond, Hybrid TPMS | **√** | ^[45]^ |
| Single | Hydrogen | Simulation | / | Gyroid, Diamond, Primitive | **√** | ^[46]^ |
| Two | Air/ CO_2_ | Simulation | / | Gyroid, Diamond, Primitive | **√** | ^[47]^ |
| Single | Air | Simulation | / | Diamond | **√** | ^[48]^ |
| Single | Air | Simulation | / | TPMS, foam | **√** | ^[49]^ |
| Single/two | Water | Simulation | / | Gyroid, Diamond,  I-WP, Primitive | **√** | ^[50]^ |
| Single | Air | Simulation | / | I-WP, Neovius, Primitive, Fischer-Koch | **√** | ^[51]^ |
| Two | Air | Simulation | / | Fischer-Koch, Gyroid, F-RD | **√** | ^[52]^ |
| Single | Water | Simulation | / | Gyroid, Diamond, Primitive, Lidinoid | **√** | ^[53]^ |
| Two | Water | Simulation | / | Gyroid, Primitive, Diamond, IWP | **√** | ^[54]^ |
| Single | Air | Simulation | / | Gyroid | **√** | ^[55]^ |

(Continue)

| **Single/Two fluid domain** | **Fluid** | **Experiment or/and simulation** | **Constituent**  **material** | **TPMS type** | **Unit-cell-level** | **References** |
| --- | --- | --- | --- | --- | --- | --- |
| Single | Air | Simulation | / | Gyroid, Primitive | **√** | ^[56]^ |
| Single | Air | Simulation | / | I-WP, Primitive, Diamond, Gyroid | **√** | ^[57]^ |
| Single |  | Simulation and experiment | Resin | Gyroid, Primitive, Diamond, IWP | **√** | ^[58]^ |
| Single/two | Water/air | Simulation and experiment | Resin | Gyroid | **√** | ^[59]^ |
| Single | Water/air | Simulation and experiment | Resin | Fischer-Koch, Diamond | **√** | ^[60]^ |
| Single | Water | experiment | Photopolymer resin | Primitive, Diamond | **√** | ^[61]^ |
| Single | H2 | Experiment | LaNi5 | Gyroid | **√** | ^[62]^ |
| Two | Water/air | Experiment | Resin | Gyroid, Diamond, Primitive, I-WP, Fischer-Koch | **√** | ^[63]^ |
| Single | Air | Simulation and experiment | Al | Gyroid, Diamond | **√** | ^[64]^ |
| Two | Water | Review and experiment | Al | Gyroid, Primitive, Diamond | **√** | ^[65]^ |
| Single | Water | Experiment and simulation | Al | Diamond | **√** | ^[66]^ |
| Single | CO_2_ | Simulation and experiment | Al | I-WP, Primitive, Gyroid, Diamond | **√** | ^[67]^ |
| Single | Air | Simulation and optimization | Al | Gyroid | **√** | ^[68]^ |
| Single | Air | Simulation and experiment | AlSi10Mg | Gyroid, Diamond | **√** | ^[69]^ |
| Two | Air/water | Simulation and experiment | AlSi10Mg | Gyroid | **√** | ^[70]^ |
| Single | Air | Simulation and experiment | Al alloy | Gyroid, Diamond, I-WP | **√** | ^[71]^ |
| Two | Air | Simulation and experiment | Al alloy | Gyroid, Diamond, Primitive | **√** | ^[72]^ |
| Two | Water | Simulation and experiment | Pure copper | Diamond | **√** | ^[73]^ |
| Two | Water | Simulation and experiment | Pure copper | Diamond | **√** | ^[74]^ |

(Continue)

| **Single/Two fluid domain** | **Fluid** | **Experiment or/and simulation** | **Constituent**  **material** | **TPMS type** | **Unit-cell-level** | **References** |
| --- | --- | --- | --- | --- | --- | --- |
| Single | Air | Simulation | / | Diamond, Gyroid | **√** | ^[75]^ |
| Single | Air | Simulation and experiment | AlSi10Mg/Phase change materials | Primitive (Anisotropic) | **√** | ^[76]^ |
| Single | Air/Hydrogen | Simulation | / | Diamond, Gyroid, Primitive | **√** | ^[77]^ |
| Single | Phase change material | Simulation and experiment | AlSi10Mg | Primitive | **√** | ^[78]^ |
| Single | Water | Simulation | Al | Primitive, Diamond, Gyroid, I-WP | **√** | ^[79]^ |
| Single | Phase change material | Simulation | AlSi10Mg | Primitive | **√** | ^[80]^ |
| Two | Water | Simulation and experiment | AlSi10Mg | Gyroid | **√** | ^[81]^ |
| Two | water | Simulation | AlSi10Mg | Gyroid | **√** | ^[82]^ |
| Single | Phase change material | Simulation | Al | Primitive, Gyroid, I-WP | **√** | ^[83]^ |
| Two | Helium/Air | Simulation | Inconel-718 | Gyroid | **√** | ^[84]^ |
| Single | water | Simulation | Titanium alloy | I-WP | **√** | ^[85]^ |
| Two | Helium/Air | Simulation and experiment | AISI 316L | Diamond | **√** | ^[86]^ |
| Single | Water | Simulation and experiment | AlSi10Mg | Diamond, Gyroid, I-WP | **√** | ^[87]^ |

# Decoding labyrinthine channels in TPMS metamaterials

## 2.1 TPMS channel characteristics: interwoven, disjoint, and labyrinthine

**S-Fig. 1** illustrates the mutually disjoint and intertwined channels within the TPMS structures. The columns 1 and 2 in **S-Fig. 1** display the solid sheet structure (in copper color) alongside the hot and cold fluid domains (in green and purple, respectively). Column 3 demonstrates how the solid sheet structures, combined with the two intertwined channels, occupy the space. **S-Table 3** lists the equations for some TPMS metamaterials. More details can refer to Section 1, Materials and Methods.

**S-Table 3 Mathematical equations describing TPMS metamaterials.**

| **TPMS metamaterials** | **Mathematical equations** |
| --- | --- |
| Fischer- Koch | cos(0.5$\times\pi\times x$)$\times$sin(0.25$\times\pi\times y$) $\times$cos(0.25$\times\pi\times z$)+cos(0.5$\times\pi\times y$) $\times$sin(0.25$\times\pi\times z$) $\times$cos(0.25$\times\pi\times x$)+cos(0.5$\times\pi\times z$)  $\times$sin(0.25$\times\pi\times x$) $\times$cos(0.25$\times\pi\times y$)+t=0 |
| Gyroid | cos(0.25$\times\pi\times x$) $\times$sin(0.25$\times\pi\times y$)+cos(0.25$\times\pi\times y$)$\times$sin(0.25$\times\pi\times z$)+cos(0.25$\times\pi\times z$) $\times$sin(0.25$\times\pi\times x$)+t=0 |
| Primitive | cos(0.25$\times\pi\times x$)+cos(0.25$\times\pi\times y$)+cos(0.25$\times\pi\times y$)+t=0 |
| Diamond | cos(0.25$\times\pi\times x$)$\times$sin(0.25$\times\pi\times y$)+cos(0.25$\times\pi\times y$) $\times$sin(0.25$\times\pi\times z$)+cos(0.25$\times\pi\times z$) $\times$sin(0.25$\times\pi\times x$)+t=0 |
| I-WP | 2$\times$cos(0.25$\times\pi\times x$) $\times$cos(0.25$\times\pi\times y$)+2$\times$cos(0.25$\times\pi\times y$) $\times$cos(0.25$\times\pi\times z$)+2$\times$cos(0.25$\times\pi\times x$) $\times$cos(0.25$\times\pi\times z$)  -cos(0.25$\times\pi\times2x$)-cos(0.25$\times\pi\times2y$)-cos(0.25$\times\pi\times2z$) +t=0 |
| F-RD | 4$\times$cos(0.25$\times\pi\times x$)$\times$cos(0.25$\times\pi\times y$) $\times$cos(0.25$\times\pi\times z$)-cos(0.25$\times\pi\times2x$)$\times$cos(0.25$\times\pi\times2y$)-cos(0.25$\times\pi\times2y$)  $\times$cos(0.25$\times\pi\times2z$)-cos(0.25$\times\pi\times2z$) $\times$cos(0.25$\times\pi\times2x$)=0 |
| Neovius | 3$\times$cos(0.25×π×x)+3$\times$cos(0.25×π×y)+3×cos(0.25×π×z)+4×cos(0.25×π×x)×cos(0.25×π×y)×cos(0.25×π×z)=0 |
| Note: Fischer-Koch structure in this work refers to Fischer-Koch S. Parameter t is used to tune the relative density of TPMS. | |

**S-Fig. 1 Solid and fluid domains of TPMS metamaterials.**

## 2.2 Crystallographic symmetry: applying skeletons to describe the labyrinthine channels

**S-Fig. 2** illustrates the topology of two sets of skeletons (marked in red and blue), representing the labyrinths of TPMS. These two sets describe the two channels of the TPMS structures and share the same topology—referred to as equilibrium channels—except for the I-WP and F-RD configurations, which will be further discussed in **S-Section 2.6.3**. Additionally, **S-Table 4** provides a summary of the space group number for the skeleton marked in red.

**S-Fig. 2 Skeletons used to depict two labyrithine channels of the selected six TPMS metamaterials.**

**S-Table 4 Space numbers of skeletons of six TPMS metamaterials.**

| TPMS metamaterials | Fischer-Koch | Gyroid | Primitive |
| --- | --- | --- | --- |
|  | 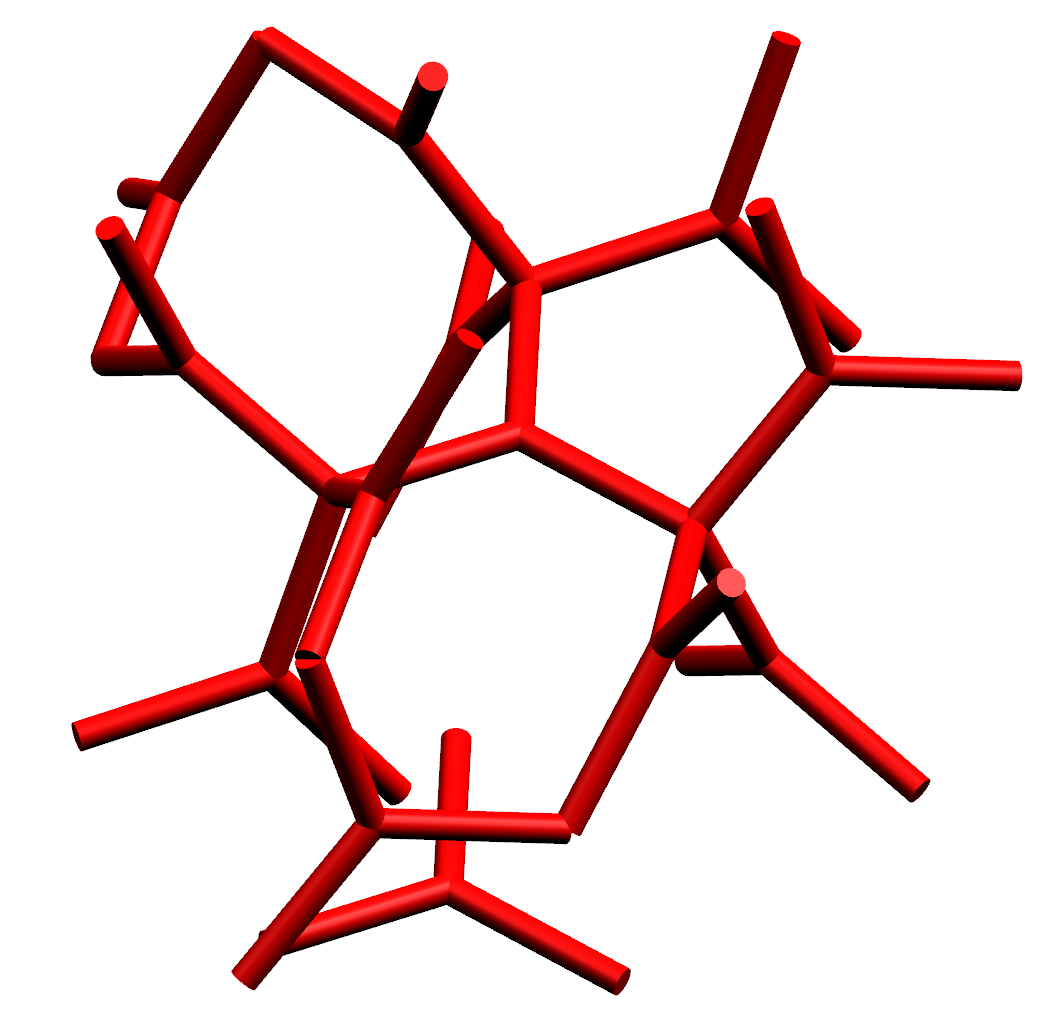 | 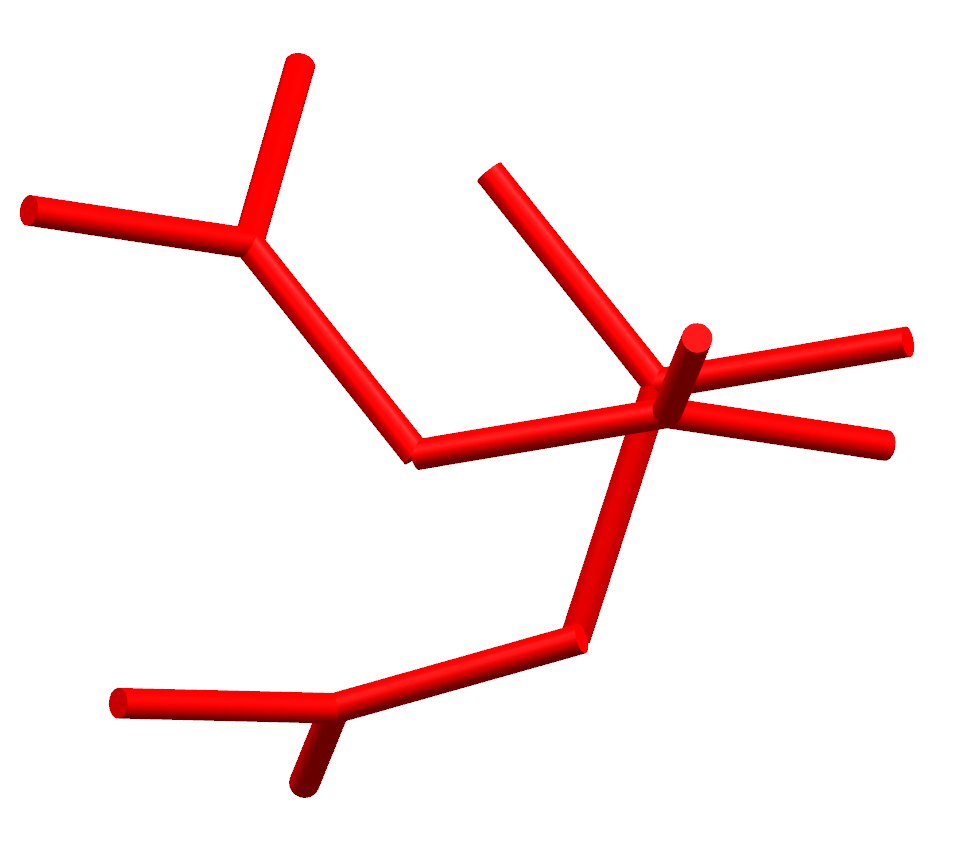 | 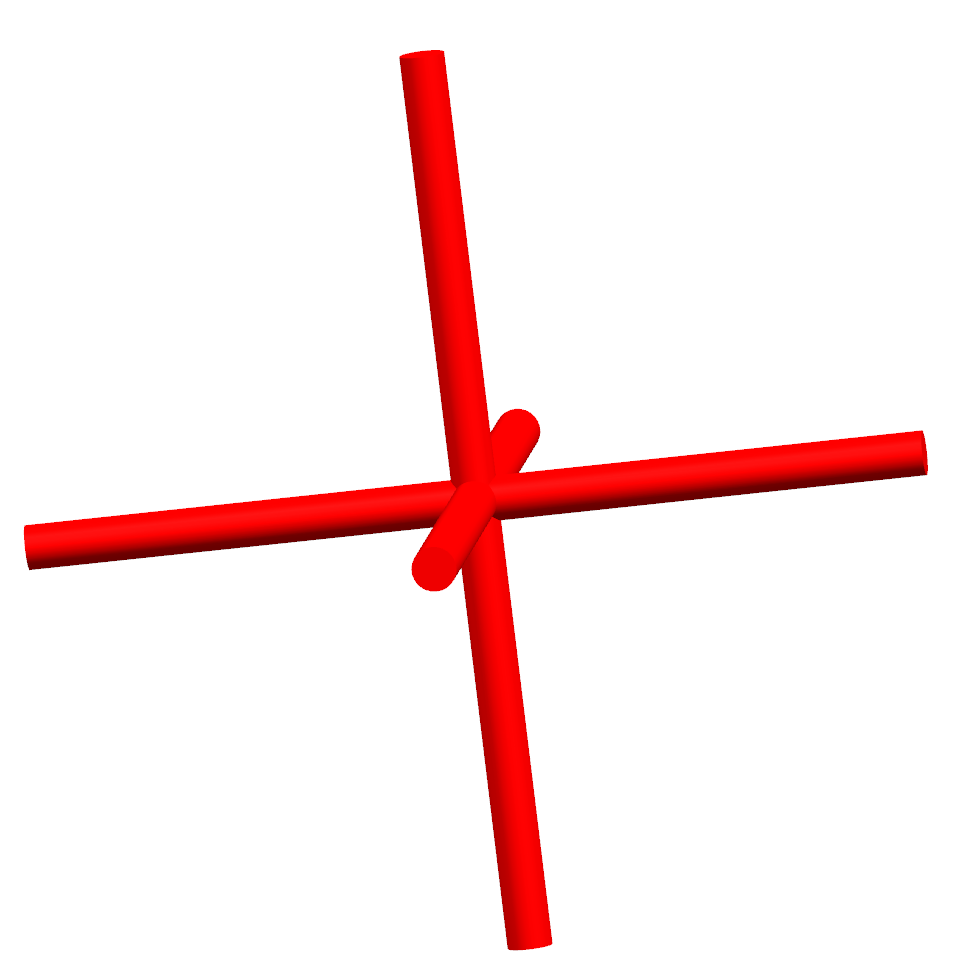 |
| Space group number | I-43d (220) | [I4_1_32](https://www.cryst.ehu.es/cgi-bin/cryst/programs/nph-getgen?list=new&what=gen&gnum=214) (214) | Pm-3m (221) |
|  | | | |
| TPMS metamaterials | Diamond | I-WP | F-RD |
|  | 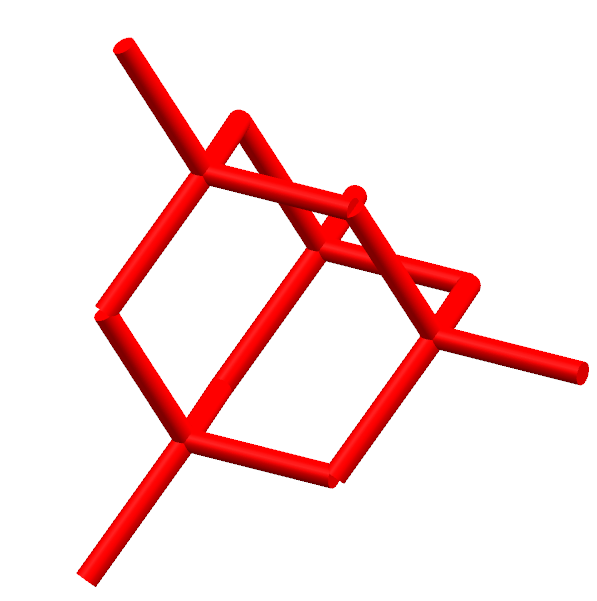 | 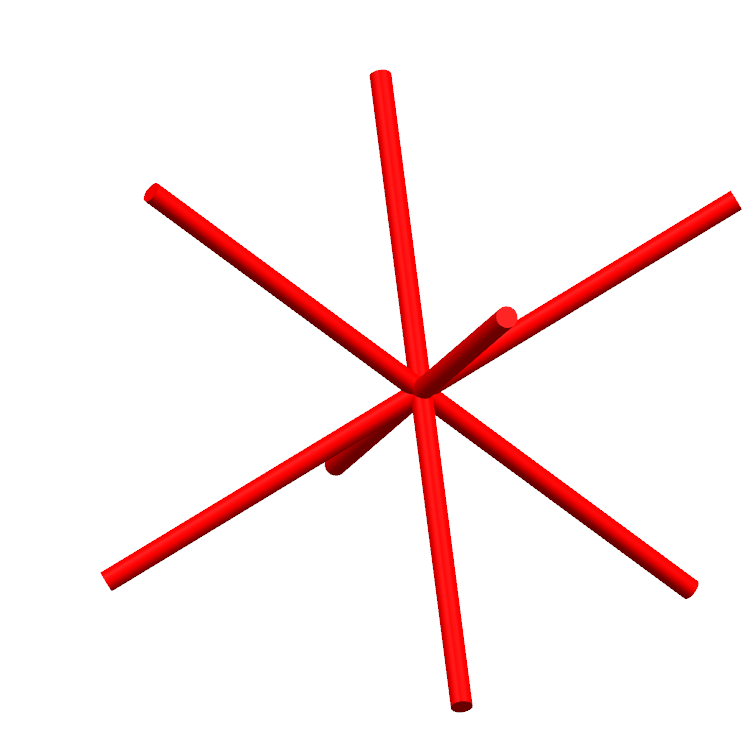 | 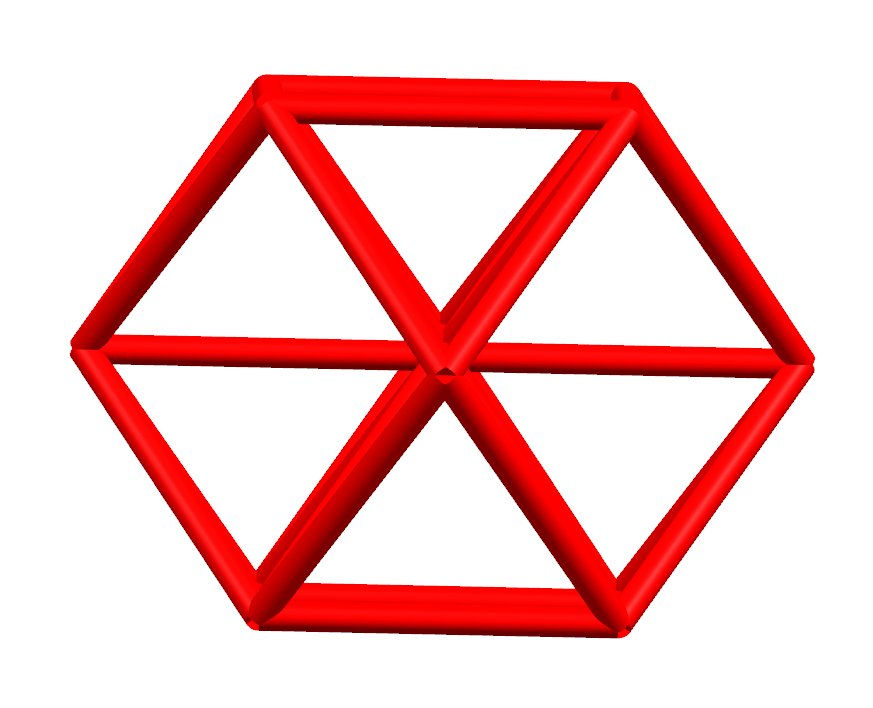 |
| Space group number | Fd-3m (227) | Im-3m (229) | Fm-3m (225) |

## 2.3. Voronoi method: extraction of the flow conduit

The flow conduit for the six selected TPMS structures is extracted through the following steps:

1. Identifying the midpoint of the skeleton of each flow conduit (the concept of the skeleton is explained in **S-Section 2.2** above);
2. Constructing the Voronoi diagram with further details provided in **S-Section 2.4.2**, where the shaded area lies within the Voronoi cell;
3. Defining the flow conduit as the portion of the TPMS structure enclosed within the Voronoi cell.

An elementary building block is a fundamental component of a space under group action. It refers to a region that, when subjected to certain transformations (typically symmetries or translations), can generate the entire space through these actions. It is noteworthy that these building blocks are not always identical:

- In the realm of geometry modeling, this building block of TPMS is referred to as the fundamental region (also known as Flächenstück) ^[88]^;
- In crystallography, it takes the form of surface patches ^[89, 90]^; while in mechanical metamaterials, it manifests as unit cells;
- In fluid dynamics, the flow conduit represents a special case, as it serves as the most fundamental unit for such evaluations.

It is also worth mentioning that the choice of unit cell for specific TPMS metamaterials is not unique (see the subsequent **S-Section 2.6** for more details). Consequently, our analysis focuses on four sub-unit-cell-informed structural descriptors of TPMS.

**S-Fig. 3** illustrates, using the Fischer–Koch topology as a representative example, how the extracted conduit-like sub-unit cell constitutes a true intrinsic building block of the TPMS architecture. Starting from a single intrinsic conduit, the complete Fischer–Koch unit cell can be reconstructed exclusively through a sequence of symmetry operations, without any geometric deformation, rescaling, or topological modification of the conduit itself. As shown, adjacent conduits are generated by successive 120° rotations, reflecting the underlying crystallographic symmetry of the Fischer–Koch structure. Repeating this rotational assembly systematically produces a total of 48 conduits with identical geometry but different spatial orientations, which together tile the full unit cell seamlessly. This explicit reconstructability confirms that the conduit-like sub-unit cell is not an arbitrary segmentation artifact, but a symmetry-consistent, geometry-invariant intrinsic unit that fully encodes the topology of the parent TPMS.

**S-Fig. 3 The Fischer–Koch TPMS unit cell is constructed step by step from a single intrinsic conduit.** A total of 48 identical conduits, arranged in varying orientations according to the crystallographic symmetry of the Fischer–Koch TPMS (the cubic space group $I\bar{4}3d$ (No. 220)), form the complete unit cell. Conduit 2 is joined to Conduit 1 by rotating Conduit 1 by 120°; Conduit 3 is joined to Conduit 2 by rotating Conduit 2 by another 120°; and Conduit 4 is joined to Conduit 3 by rotating Conduit 3 by 120°. Repeating this rotational assembly process systematically generates the full Fischer–Koch TPMS unit cell comprising 48 conduits.

## 2.4 The surface area-to-volume ratio

The definitions of surface area-to-volume ratio are summarized in **S-Table 5**. In the main text, we use definition 6.

**S-Table 5 Different definitions of surface area-to-volume ratio.**

| Definitions | | Scale-variant | Scale-invariant |
| --- | --- | --- | --- |
| 1 |   Unit cell | $S_{\text{Red+Blue}}/V_{\text{Solid}}$ | $S_{\text{Red+B}\text{lue}}/{(V_{\text{Solid}})}^{2/3}$ |
| 2 |  | ${S_{\text{Red or blue}}}/{V_{\text{Solid}}}$ | $S_{\text{Red or B}\text{lue}}/{(V_{\text{Solid}})}^{2/3}$ |
| 3 |  | $S_{\text{Red+B}\text{lue}}/V_{\text{Gr}\text{ay}\text{ cubic}}$ | $S_{\text{Red+B}\text{lue}}/{(V_{\text{Gr}\text{ay}\text{ cubic}})}^{2/3}$ |
| 4 |  | ${S_{\text{Red or blue}}}/{V_{\text{Gr}\text{ay}\text{ cubic}}}$ | $S_{\text{Red or B}\text{lue}}/{(V_{\text{Gr}\text{ay}\text{ cubic}})}^{2/3}$ |
| 5 | 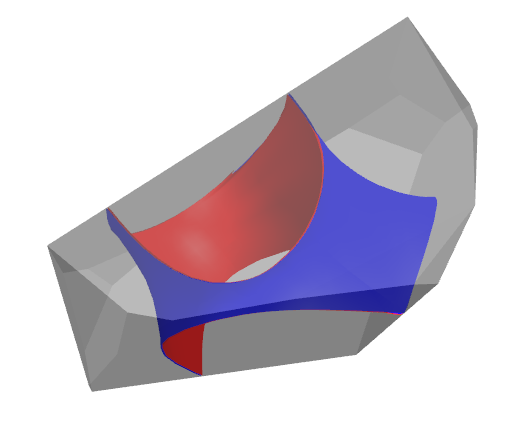  Flow conduit | $S_{\text{Red}\text{ + }\text{B}\text{lue}}/V_{\text{Gr}\text{ay}\text{ cubic}}$ | $S_{\text{Red+B}\text{lue}}/{(V_{\text{Gr}\text{ay}\text{ cubic}})}^{2/3}$ |
| 6 |  | $S_{\text{Red}\text{ or }\text{B}\text{lue}}/V_{\text{Gr}\text{ay}\text{ cubic}}$ | $S_{\text{Red}\text{ or }\text{B}\text{lue}}/{(V_{\text{Gr}\text{ay}\text{ cubic}})}^{2/3}$ |

## 2.5 The flow conduit diameter

Calculating the flow conduit diameter in TPMS structures is more complex than in cylinder-based heat exchangers. To address this complexity, the Voronoi method is applied. Voronoi partitioning divides space into regions, or cells, around a set of geometric objects (typically points). Each region is defined so that every point within it is closer to its associated object than to any other object in the set, as illustrated in **S-Fig. 4**.

**S-Fig. 4**a illustrates how the Voronoi structure is applied to calculate channel diameters. The polygons, marked in different colors, represent the Voronoi cells corresponding to the black points within them. The Voronoi structure divides the flow conduits into several sections, and we calculate the diameter of each section, as indicated by the white arrows. **S-Fig. 4**b provides a 3D visualization of the diameter distribution in a Primitive TPMS channel. The TPMS flow conduits diameter is calculated based on the Voronoi method, which partitions space using the vertex points that form the Voronoi cells, ensuring that the distance from any point in the Voronoi cell to the vertex point is minimized. The diameter is calculated as follows:

- The points in the TPMS structure (specifically, the vertices of the triangles in the obj or stl file are treated as vertex points;
- The distance from each vertex within the corresponding Voronoi cell is approximated as the flow conduit diameter, denoted as $d$ ($d_{1}, d_{2}, d_{3}, d_{4},\ldots$). The diameter distribution is consistent for two equilibrium flow conduits in TPMS structures, while it varies in non-equivalent channels within TPMS (see definition in **S-Section 2.6.3**).

**S-Fig. 4 Methods for identifying the TPMS flow conduit diameter.** (a) Schematic illustration of the diameter represented by Voronoi structures, adapted from the Interactive Voronoi Diagram ^[91]^. (b) Diameter distribution determined by the Voronoi-defined spheres in TPMS structure, using Primitive TPMS as an example.

Using this method, we can calculate the diameter of the TPMS structures. To express this in a dimensionless form, we utilized the coefficient of variation ($\bar{d}/\sigma$), where $\bar{d}$ is the mean diameter of the TPMS and $\sigma$ is the standard deviation. **S-Fig. 5** illustrates the diameters of the selected TPMS structures.

**S-Fig. 5 Flow conduit diameters of the selected six TPMS structures.** $\bar{d}$: the mean diameter,$\sigma$: the channel diameter standard deviation.

While the volumetric hydraulic diameter D_h_=4εV/A ^[54, 92-94]^ (ε: porosity, V: the three-dimensional volume of unit cell, A: the wetted surface area) provides a meaningful global length scale, most TPMS architectures exhibit highly tortuous and intrinsically non-uniform flow channels, characterized by strong spatial variations in local cross-section, necking, and branching. To explicitly quantify this non-uniformity, we introduce the conduit uniformity metric
$d$/σ, where d is the mean Voronoi-based diameter and σ its standard deviation along the interconnected channel network (as will be shown in **S-Table 9**). Using a conservative threshold for relatively uniform channels, we find that only 5 out of the 27 representative TPMS structures analysed in this work satisfy this criterion (**Fig. 4b**), indicating that the majority (22/27) possess pronounced intrinsic diameter non-uniformity. Under such conditions, a single global hydraulic diameter inevitably averages out local constrictions that dominate flow resistance, whereas the Voronoi-based diameter provides a spatially resolved description of the channel diameter distribution and is therefore more representative for most TPMS architectures considered here. For illustration, **S-Table 6** compares the two diameter measures for a Primitive TPMS with porosity $\varepsilon=0.9$. The results highlight how the two approaches lead to markedly different interpretations of the same flow geometry, with the Voronoi framework more faithfully representing the underlying geometric variation.

**S-Table 6** Comparison between the volumetric hydraulic and Voronoi diameters for a Primitive TPMS with ε=0.9 under identical geometric conditions.

| Hydraulic diameter (ε=0.9) | Voronoi-based diameter (ε=0.9) | | | | |
| --- | --- | --- | --- | --- | --- |
|  | d_max_ | d_min_ | $d$ | $\sigma$ | $d$/$\sigma$ |
| 5.6  ($\sigma$ = 0) | 6.5 | 1.5 | 4.8 | 1.3 | 3.69 |

## 2.6 Euler index

The Euler index is dependent on the Gaussian curvature, and the Euler index referenced in this manuscript corresponds to a flow conduit. **S-Table 7** summarizes the exterior angles and Euler indices for the selected six TPMS structures.

**S-Table 7 External-angle values and Euler index of the flow conduits of selected six TPMS metamaterials.**

| TPMS | Fisher-Koch | Gyroid | Diamond | I-WP | F-RD | Primitive |
| --- | --- | --- | --- | --- | --- | --- |
| Sum of external angle ($\sum\theta$) | 5π/3 | 4π/3 | 2π | 3π | 5π/2 | 8π/3 |
| Euler index  $\left\vert\chi\right\vert$ | 0.83 | 0.67 | 1.00 | 1.50 | 1.25 | 1.33 |

As previously noted, the flow conduit serves as the fundamental thermal-related building block. Meanwhile, the unit cell, particularly in its cuboid form, is a crucial metric in both design and manufacturing. It is logical to scale indicators such as ($N$, $S/{V^{2/3}}$, $\bar{d}/\sigma$, $\left| \chi\right|$) to the unit cell level.

The relationship between the unit cell and flow conduit with respect to $\left| \chi\right|$, $S/{V^{2/3}}$ and $\bar{d}/\sigma$ is described by **S-Eqs. 3-8**. By incorporating $N^{1/3}$ , ξ represents the characteristic of the unit cell, underscoring its importance in design and manufacturing. Similarly, by incorporating N, |χ|×N represent the overall connectivity smoothness of the unit cell. As for flow conduit spatial density, N refers specifically to the number of skeleton lines within a unit cell.

| $\left\vert\chi\right\vert_{\text{Unit cell}}={N\times\left\vert\chi\right\vert}_{\text{Flow conduit}}$ | **(3)** |
| --- | --- |
| ${(\bar{d}/\sigma)}_{\text{Unit cell}}={(\bar{d}/\sigma)}_{\text{Flow conduit}}$ | **(4)** |

The relationship between the unit-cell and conduit-level specific surface area can be derived analytically. For a TPMS unit cell composed of N geometrically equivalent and independent flow conduits, the total surface area and volume scale as

| $S_{\text{Unit cell}}=N\times S_{\text{Flow conduit}}$ | **(5)** |
| --- | --- |
| $V_{\text{Unit cell}}={N\times V}_{\text{Flow conduit}}$ | **(6)** |
| $\left[ S/{V^{2/3}} \right]_{\text{Unit cell}}={N\times S_{\text{Flow conduit}}}/{{({N\times V}_{\text{Flow conduit}})}^{2/3}}$ | **(7)** |

Accordingly, the unit-cell specific surface area becomes

| $\left[ S/{V^{2/3}} \right]_{\text{Unit cell}}=N^{1/3}{\times\left[ S/{V^{2/3}} \right]}_{\text{Flow conduit}}$ | **(8)** |
| --- | --- |

This result shows that the unit-cell specific surface area scales with the conduit-level value through a N^1/3^ factor, reflecting the three-dimensional packing of multiple conduits rather than a simple linear superposition.

## 2.7 The reducible mapping among TPMS descriptors

**S-Fig. 6 Descriptor reduction based on sub-unit-cell topology.** Comprehensive TPMS metrics—geometric or topological—are shown to be reducible to four sub-unit-cell variables (N, S/V²/³, d̅/σ, |χ|), establishing a compact and sufficient descriptor set for transport analysis.

**S-Table 8 External-angle values and Euler index of the flow conduits of selected six TPMS metamaterials.**

| Metric | Reduction Formula | Reducible Parameter |
| --- | --- | --- |
| Surface area S | Defined in S/V²/³ | S/V²/³ |
| Volume V | Defined in S/V²/³ | S/V²/³ |
| Unit size L | V = L³ ⇒ S/V²/³ = S/L² | S/V²/³ |
| Relative density ρ_RD_ | S = S(ρ_RD_) ⇒ S/V²/³ | S/V²/³ |
| Pore size d_pore_ | dᵢ ≈d_pore_ ⇒ d̅ ∼d_pore_ | d̅/σ |
| Wall thickness t | dᵢ ≈d_pore_ − t ⇒ d̅ = ⟨dᵢ⟩ | d̅/σ |
| Channel cross-section Aᵢ | dᵢ = 2√(Aᵢ/π), defines d̅, σ | d̅/σ |
| Channel diameter dᵢ | d̅ = ⟨dᵢ⟩, σ = std(dᵢ) | d̅/σ |
| Channel connectivity C | C ≈ N⟨z⟩ ⇒ C ∝ N | N |
| Channel density N | N | N |
| Skeleton node degree zⱼ | Σⱼ zⱼ = 2E, N ∝ Σⱼ zⱼ | N |
| Space group | Determines multiplicity of nodes/edges ⇒ N | N |
| Wyckoff position | Defines orbit multiplicity ⇒ N, χ | N, \|χ\| |
| Symmetry operation | Generates repetition, thus N and χ | N, \|χ\| |
| Mean curvature Ĥ | Ĥ ≈ 0 for TPMS | \|χ\| |
| Gaussian curvature K | Gauss–Bonnet: ∫K dA = 2πχ | \|χ\| |
| Genus g | χ = 2–2g ⇒ g ↔ χ | \|χ\| |
| Topology index T | T = (S/V²/³/(2π) \|χ\|)^0.5^ | \|χ\| |
| **Note: i**: index for individual conduit / channel element inside a sub-unit cell; j: index for skeleton nodes (junction points) within the skeletal network | | |

## 2.8 Physically grounded geometric screening of TPMS architectures

In our framework, $\bar{d}/\sigma$ and $\xi=S/V^{2/3}\times N^{1/3}$ are two dimensionless geometric descriptors expected to significantly affect the TPMS heat-exchange performance:

$\bar{d}/\sigma$— a channel-uniformity indicator (more details are provided in **S-Table 9** below)

$\xi$— a packing-limited surface density (see Supplementary Eqs. 5–8). This parameter quantifies how much heat-exchange surface is available per unit of packing density.

Based on principal component analysis (PCA), we adopt $\bar{d}/\sigma$ as the primary screening parameter, with $(\bar{d}/\sigma)\times\xi$ used as a secondary ranking metric. To ensure that the screening criterion is physically transparent rather than arbitrary, **S-Table 9** maps the diameter variation ratio $\sigma/d$ (or its reciprocal $d/\sigma$) to channel morphologies with direct geometric interpretation. The classification is**referenced against** two considerations: (i) the approximate human visual detection limit for diameter variation under typical viewing conditions (≈ 0.3 mm), which provides a perceptual reference scale; and (ii) systematic geometric visualizations generated using a scripted MATLAB model in which $\sigma/d$ was continuously varied from 1 to 50, revealing progressive transitions in channel morphology, including necking, surface roughness, and geometric discontinuity.

**S-Fig. 7**  presents representative visualizations of this classification, with cross-sectional (left), plan (middle), and three-dimensional (right) views corresponding to the entries in **S-Table 9**. Together, these criteria establish practical, visually interpretable thresholds for channel uniformity rather than strict physical boundaries.

**S-Table 9** Channel uniformity based on the diameter variation ratio $\sigma/d$ (or equivalently $d/\sigma$), calibrated against visually resolvable geometric features and and the human eye detection limit (≈0.3 mm).

| Variation  ($\sigma/\bar{d}$) | Uniformity ratio ($\bar{d}/\sigma$) | Hydraulic and thermal implication |
| --- | --- | --- |
| 100% | 1 | Chaotic geometry with severe choking and partial disconnection |
| 50% | 2 | Strong necking and geometric fragmentation |
| 33% | 3 | Poor to moderate uniformity with large cross-sectional fluctuations |
| 25% | 4 | Very rough channels with clearly visible necking |
| 20% | 5 | Generally acceptable; continuous channels with weak necking |
| 10% | 10 | Fairly uniform flow paths with negligible necking |
| 5% | 20 | Near-ideal geometry with negligible geometric disturbance |
| 0% | ∞ | Perfectly cylindrical channel with a uniform cross-section |


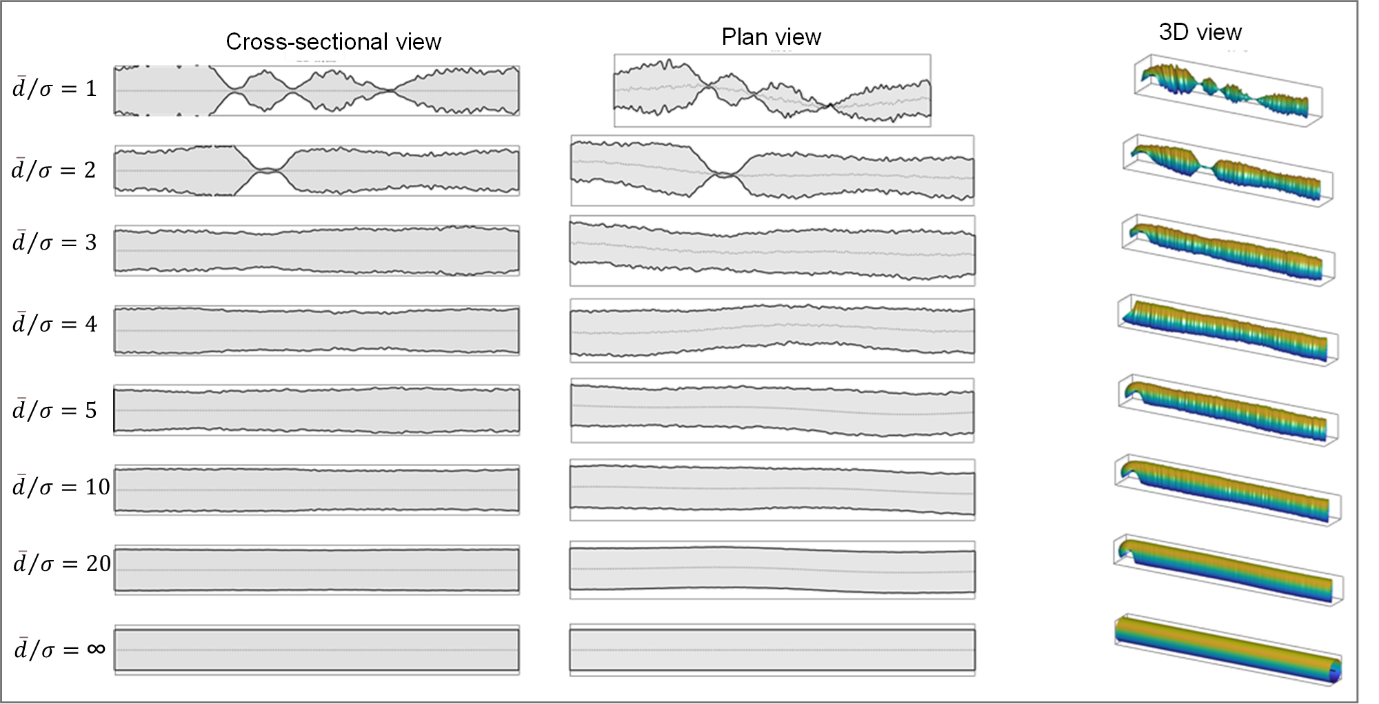


**S-Fig. 7** Geometric manifestations of channel non-uniformity across different σ/d levels. left: a 2D cross-sectional view, middle: a 2D plan view, right: a 3D perspective tube rendering.

## 2.9 Other topology considerations for transport

This section discusses other topological factors, including unit cell size, anisotropy, and symmetry balance between the flow conduits. These considerations further justify the rationale behind focusing on flow conduit analysis in the main text.

### 2.9.1 The unit-cell selection

The unit cell of TPMS metamaterials is not unique. We take the diamond TPMS as an example to illustrate it. **S-Fig. 8** presents two options for unit cells of the diamond metamaterial. For the unit cell shown in **S-Fig. 8**a, which is hexahedron-like, repeating it along the directions indicated by the red arrows generates a three-dimensional diamond metamaterial. Similarly, the unit cell depicted in **S-Fig. 8**b, which is cuboid-like, can also be repeated along the directions indicated by the red arrows to produce a three-dimensional diamond metamaterial.

In summary, the physical properties of TPMS metamaterials, including heat exchange ability, depend on the number, size, and selection of unit cells ^[92, 96]^. Therefore, to compare TPMS structures, it is essential to consider quantities that are not only dimensionless but also independent of the choice of unit cell. This is why we use four dimensionless parameters related to the flow conduit rather than the “unit cell” to analyze thermal performance.

**S-Fig. 8 Two definitions of unit cells of diamond metamaterials.** (a) hexahedron-like unit cell. (b) cuboid-like unit cell.

### 2.9.2 Preliminary evaluation of anisotropy

The results in **S-Fig. 9** indicate that the heat transfer rates are 4.26 W in the direction [111] and 4.09 W in the direction [001], showing a relative difference of 3.99%. Additionally, the temperature differences between the inlet and outlet are 5.25°C for the direction [111] and 4.80°C for the [001] direction, resulting in a relative difference of 8.57%. These findings suggest that the direction of fluid flow at the inlet and outlet influences the heat transfer performance of TPMS structures, although the extent of this effect has not been systematically analyzed. Therefore, when comparing the heat transfer efficiency of different TPMS designs, it is necessary to consider the inlet and outlet orientations carefully. In this study, we default to the direction [001], and the quantitative effects of anisotropy will be explored further in future work.

**S-Fig. 9 Fluid flow in and out directions in diamond metamaterial.** (a) Definition of flow direction. (b-c) Fluid flow in and out along the direction [111]. (d-e) Fluid flow in and out along the direction [001].

### 2.9.3 The non-equivalent channel

The channels corresponding to the hot-fluid and cold-fluid pathways in the F-RD and I-WP structures are non-equivalent (different), while the Primitive structure exhibits pseudo-non-equivalent behavior (see **S-Fig. 10** and **S-Fig. 11**), which will be discussed below.

**F-RD and I-WP**: The two channels in the F-RD structure are non-equivalent, as illustrated in **S-Fig. 10** a-c. This results in differing diameter distributions (**S-Fig. 10** d-e) and a significant pressure drop (**S-Fig. 10** f-g) across these two channels of the F-RD. The I-WP TPMS also displays a similar non-equivalent phenomenon (**S-Fig. 10** h-m).

**S-Fig. 10 Analysis on non-equivalent channels of F-RD and I-WP.** ( a-c, h-j) Non-equivalent structures of F-RD and I-WP, respectively. (d-e, k-l) Diameter distribution of the non-equivalent structures of F-RD and I-WP, respectively. (f-g, m) Simulated (and experimental) pressure drop measurements for F-RD and I-WP, respectively.

**Primitive**: The two channels of the Primitive structure are in equilibrium in terms of topology, as shown in **S-Fig. 11** A-C, which differs from the F-RD and I-WP structures. However, it is unexpected that the Primitive exhibits a significant difference in pressure drop between the two channels, with one side experiencing nearly five times the pressure drop of the other. This “pseudo non-equivalent phenomenon” may arise from the fact that the contact area of the Primitive TPMS unit cell with the outer side is closely related to the selection of periodicity and the number of unit cells. The observed relationship between the flow and heat transfer of Primitive is mainly influenced by the phase of its structure. Taking the schematic diagram in **S-Fig. 11** as an example, the red skeleton, representing the fluid within a control volume, exhibits lower resistance pressure drop when flowing into the cavity from one side and out the other. In contrast, the blue skeleton, which represents the fluid with the same inlet and outlet directions, shows higher resistance. If we consider an infinitely large volume, neglecting the resistance from the inlets, outlets, and walls, and disregarding the influence of the flow direction, the resistances on both sides would tend to equalize. This is the characteristic of “pseudo non-equivalent phenomenon” as we understand it.

**S-Fig. 11 Pseudo-non-equivalent analysis of I-WP.** (a-c) skeletons describing the channel configurations and the (d-e) corresponding tested pressure drop.

### 2.9.4 The wall thickness of TPMS

**S-Table 10** Variation of surface area with wall thickness or relative density for the Fischer–Koch TPMS (the unit-cell size is fixed at L = 10 mm).

| Relative density | Topology  (Fischer-koch, Unit size: 10 mm) | Surface area (mm^2^) |  | Relative density | Topology  (Fischer-koch, Unit size: 10 mm) | Surface area (mm^2^) |
| --- | --- | --- | --- | --- | --- | --- |
| 10% | 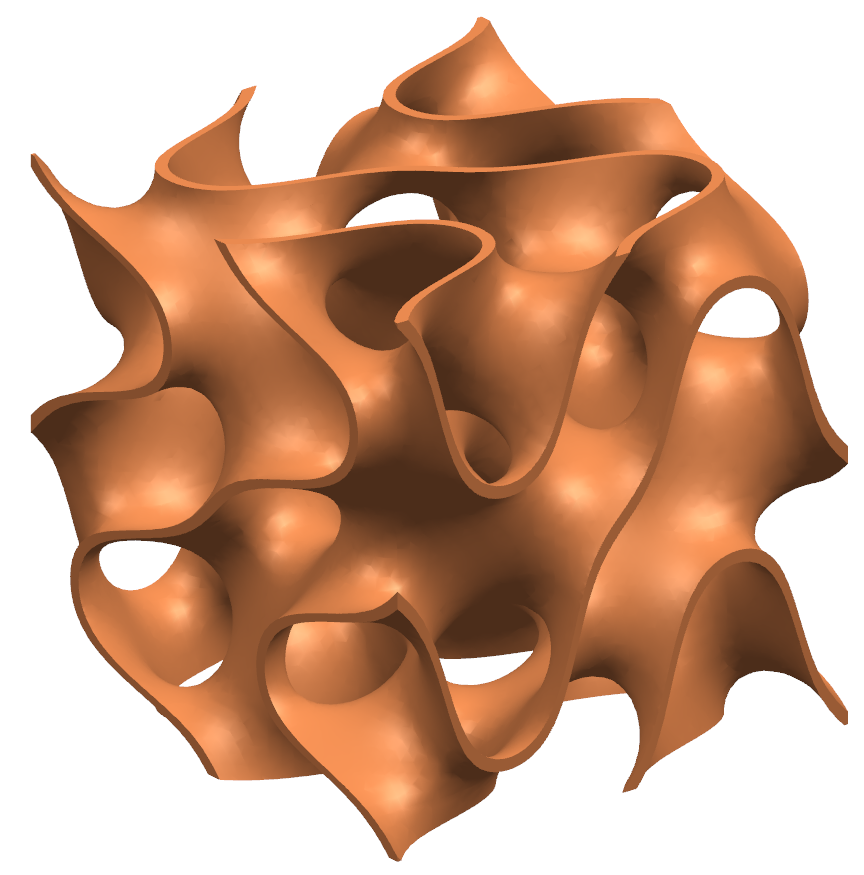 | 1079.6 |  | 30% | 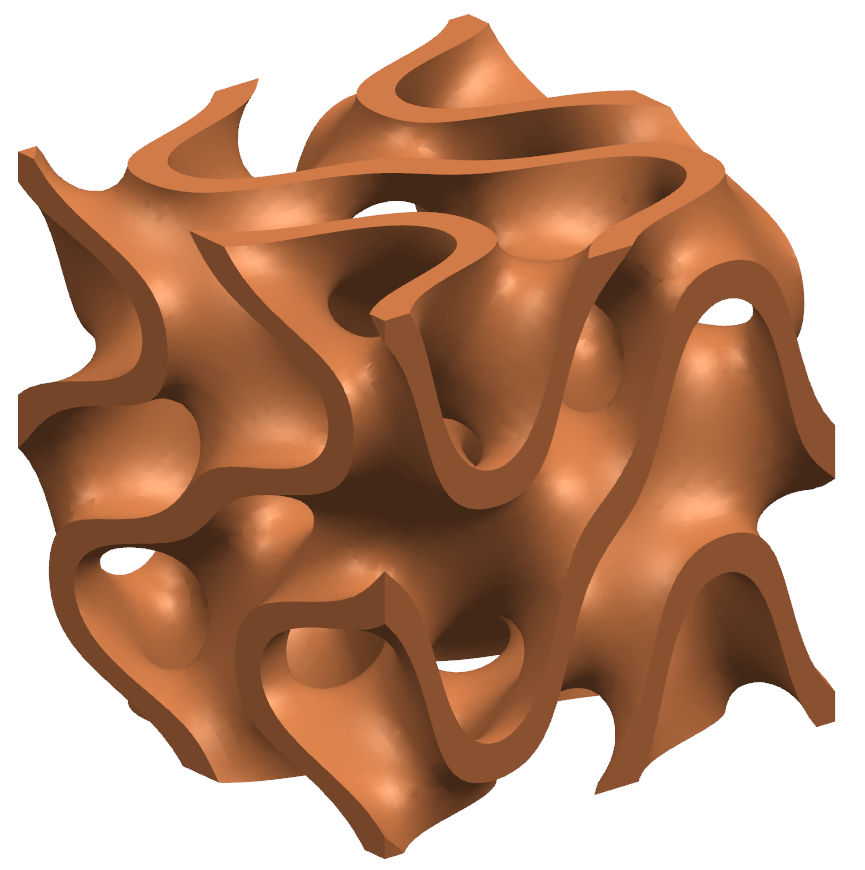 | 1039.0 |
| 20% | 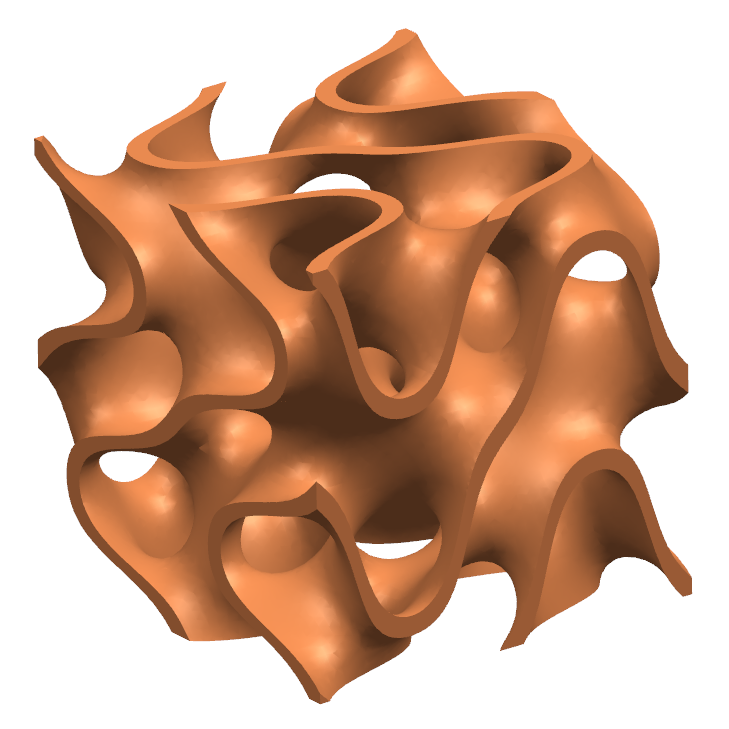 | 1064.8 |  | 40% | 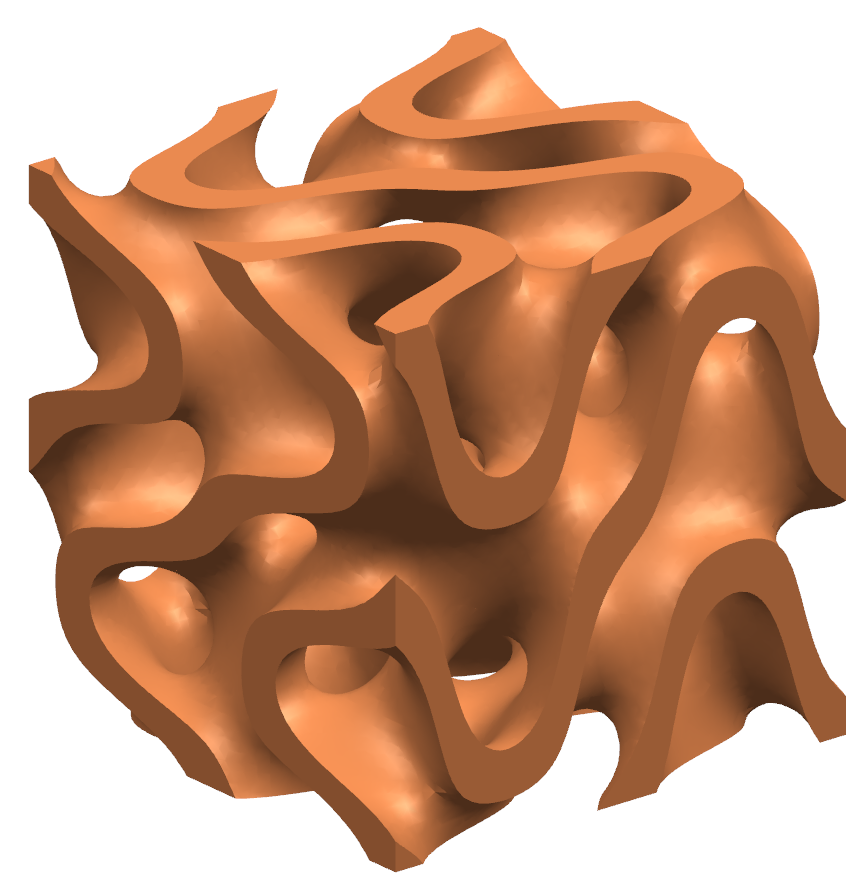 | 1000.4 |

## 2.10 η-based ranking analysis

Transport performance is evaluated by ranking the six structures based on their efficiency scores η, following the rule: ηᵢ > ηⱼ ⇒ Rankᵢ < Rankⱼ, where Rank ∈ {1st, 2nd, …, 6th} and 1st denotes the highest-performing structure. Rankings are assigned in descending order of η, such that greater η values correspond to superior (lower-numbered) performance ranks.

We performed a comprehensive parameter sweep across the exponent space, with *m, n, p, q* ∈ [0, 10] at a resolution of 0.2, generating 6.76 million unique combinations. For each combination, the performance quotient *η* was computed for six representative TPMS designs using their intrinsic descriptors (**Fig. 5a-d**), producing a ranking frequency distribution for each structure. The aggregated results (**S-Fig. 12**a) reveal a strikingly stable performance hierarchy: Fischer–Koch, Gyroid, and Diamond overwhelmingly dominate the top rankings, with Fischer–Koch consistently occupying first place. I-WP, F-RD, and Primitive form a distinct, lower-performing tier, with Primitive emerging as the least efficient.

To test the robustness of the performance hierarchy, we conducted two additional parameter sweeps: (i) exponents in [0, 10] with a step size of 0.5 (**S-Fig. 12**b), and (ii) exponents in [0, 6] with a step size of 0.2 (**S-Fig. 12**c). The ranking remained stable across both scans, with only minor fluctuations at the boundaries of the exponent space. This global sensitivity analysis confirms that the performance hierarchy is an intrinsic property of the TPMS geometries, not an artifact of specific parameter choices.

**S-Fig. 12 Robustness validation of the gene-level performance analysis.** (a) Full scan with m,n,p,q∈[0,10], step 0.2 (from main text). (b) Reduced range [0,6], step 0.2. (c) Range [0,10], step 0.5. Consistent ranking trends across all cases confirm the robustness of the framework.

## 2.11 Spearman rank correlation analysis

To quantify ranking fidelity, Spearman’s rank correlation coefficient (*ρ*) was computed between the simulated *j/f*-based ranking and the *η′*-predicted ranking at each Reynolds number. Specifically, for the n=6 TPMS architectures, we denote the simulated ranks as $\rho=1-\frac{6\sum_{i=1}^{n} d_{i}^{2}}{n(n^{2}-1)}$ ^[97]^. For completeness, ρcan equivalently be computed as the Pearson correlation between the two rank vectors. We report ρ for each Reynolds number to assess the monotonic consistency of the predicted performance hierarchy.

As comparators to **Eq. 2**, several alternative formulations were evaluated. Starting from S-Eq.9 (**Eq. 2** in the main text),

| $\eta^{'}\propto(d/\sigma)\text{ }N$ | (9) |
| --- | --- |

Replacing $N$ in S-Eq.9 with $S/V^{2/3}$gives S-Eq.10

| $\eta^{''}\propto(d/\sigma)\text{ }(S/V^{2/3})$ | (10) |
| --- | --- |

Replacing $d/\sigma$ in S-Eq.10 with $S/V^{2/3}$gives S-Eq.11

| $\eta^{'''}\propto(S/V^{2/3})\text{ }N$ | (11) |
| --- | --- |

Finally, incorporating $S/V^{2/3}$ into S-Eq.11 yields S-Eq.12

| $\eta^{''''}\propto(d/\sigma)\text{ }(S/V^{2/3})\text{ }N$ | (12) |
| --- | --- |

The results are summarized in **S-Table 11** below. η″ (**S-Eq.10**) and η‴ (**S-Eq.11**) produce rankings that deviate markedly from the simulated j/f performance trend, whereas η⁗ (**S-Eq.12**) yields rankings consistent with experimental observations. This demonstrates that removing either conduit descriptor disrupts the predicted ordering, while incorporating all descriptors preserves it.

**S-Table 11** j/f-based rankings and descriptor-predicted rankings (S-Eqs. 9–12), with Spearman’s rank correlation coefficients (ρ). Rankings are shown for three representative Reynolds numbers; see Fig. 7a for the full Re range.

| TPMS | $\text{Rank}_{\text{j/f}}$ | | | Ranking by S-Eq.9 | *ρ* | Ranking by S-Eq.10 | *ρ* | Ranking by S-Eq.11 | *ρ* | Ranking by S-Eq.12 | *ρ* |
| --- | --- | --- | --- | --- | --- | --- | --- | --- | --- | --- | --- |
|  | Re =297 | Re =2970 | Re =8910 |  |  |  |  |  |  |  |  |
| Fischer-Koch | 1 | 1 | 1 | 1 | 1.0 | 3 | 0 | 1 | 0.5 | 1 | 1.0 |
| Gyroid | 2 | 2 | 2 | 2 | 0.9 | 1 | 0.9 | 5 | 0.3 | 2 | 0.9 |
| Diamond | 3 | 3 | 3 | 3 | 1.0 | 2 | 0.9 | 3 | 0.5 | 3 | 1.0 |
| I-WP | 4 | 4 | 5 | 5 | 1.0 | 4 | 0.9 | 4 | 0.5 | 5 | 1.0 |
| F-RD | 5 | 5 | 6 | 4 | 0.8 | 6 | 0.8 | 2 | 0.1 | 4 | 0.8 |
| Primitive | 6 | 6 | 4 | 6 | 0.8 | 5 | 0.8 | 6 | 0.7 | 6 | 0.8 |
| ρ: Spearman’s rank correlation coefficient, where ρ = 1 denotes perfect agreement | | | | | | | | | | | |

# Design and manufacturing of TPMS metamaterials and heat exchangers

We elaborately utilize the state-of-art technology, GL-PBF, to manufacture the copper TPMS structures. The copper powder exhibits good sphericity (**S-Fig. 13**a) and has a particle size of D90 = 40.3 μm (**S-Fig. 13**b), which meets the requirements for laser-based powder bed fusion. Such copper powder is utilized to test the energy absorption, which increases from 21.45% by infrared laser to 63.78% by green laser (**Fig. 7 a**). These results indicate that the green laser can significantly enhance the additive manufacturability of copper.

**S-Fig. 13 Copper powder characterization.** (a) Morphologyand sphericity**.** (b) distribution of powder size.

**S-Table 12** lists the CAD parameters of the designed TPMS structures, while **S-Fig. 14** display the CAD models and corresponding printed samples. For the six selected TPMS metamaterials evaluated using μ-CT (highlighted in light blue in **S-Table 12**), the unit size is set at 10 mm with a relative density of 10%. This configuration ensures that the TPMS wall thickness exceeds the minimum printable size of GL-PBF, i.e., 500 μm, while maintaining a relatively high surface area-to-volume ratio.

On the other hand, the mechanical properties of TPMS metamaterials (highlighted in light orange in **S-Table 12**) were evaluated using structures with a relative density of ≈38%. This smaller unit size and higher relative density ensure printability while reducing the overall printing workload. **S-Table 12** summarizes the design parameters of TPMS copper metamaterials used in this work.

**S-Table 12 Design parameters of TPMS metamaterials.**

| Test items | TPMS | Unit size ($\text{mm}^{3}$) | $t_{1}$ | $t_{2}$ | Relative density | Unit-cell arrays | Three-dimensional size ($\text{mm}^{3}$) |
| --- | --- | --- | --- | --- | --- | --- | --- |
| CT and microstructure characterization  (**S-Fig. 14** | Fischer- Koch | $10\times10\times1$0 | +0.1 | -0.1 | 10.0% | $3\times3\times3$ | $30\times30\times3$0 |
|  | Gyroid | $10\times10\times1$0 | +1.5 | -1.5 | 9.7% | $3\times3\times3$ | $30\times30\times3$0 |
|  | Primitive | $10\times10\times1$0 | +0.175 | -0.175 | 10.0% | $3\times3\times3$ | $30\times30\times3$0 |
|  | Diamond | $10\times10\times1$0 | +0.13 | -0.13 | 10.7% | $3\times3\times3$ | $30\times30\times3$0 |
|  | I-WP | $10\times10\times1$0 | +0.4 | -0.4 | 10.6% | $3\times3\times3$ | $30\times30\times3$0 |
|  | F-RD | $10\times10\times1$0 | +0.25 | -0.25 | 10.8% | $3\times3\times3$ | $30\times30\times3$0 |
|  |  |  |  |  |  |  |  |
|  |  |  |  |  |  |  |  |
| Evaluation of liquid-cooling performance, **S-Fig. 14** | Fischer- Koch | $10\times10\times10$ | +0.1 | -0.1 | 10.0% | $9\times6\times3$ | $9.5\times60.5\times30.5$2 |
|  | Gyroid | $10\times10\times10$ | +1.5 | -1.5 | 9.7% | $9\times6\times3$ | $90.5\times60.5\times30.5$ |
|  | Primitive | $10\times10\times10$ | +0.175 | -0.175 | 10.0% | $9\times6\times3$ | $90.5\times60.5\times30.5$ |
|  | F-RD | $10\times10\times10$ | +0.25 | -0.25 | 10.8% | $9\times6\times3$ | $1\times60.5\times30.5$2 |

**S-Fig. 14 CAD models of TPMS metamaterials.** Detail can be found in **S-Table 12**.

**S-Fig. 15** presents the CT analysis of the printed Fischer-Koch-based heat exchangers, with the CAD model, respectively. The cross-section is uniform and continuous, indicating excellent additive manufacturability in high-field conditions.

**S-Fig. 15 Characterization of printed Fischer-Koch-based heat exchangers.** (a) μ-CT reconstructed heat exchanger model. (b) Cross-sectional views from μ-CT reconstructed heat exchanger.

# Liquid-Cooling Evalutaion of Testing of Prototypes

## 4.1 The simulation analysis

**S-Fig. 16**, **S-Table 13** and **S-Table 14** describe the simulated parameters for the liquid-cooled system. A comprehensive explanation of these simulations is provided in Section Materials and Methods.

**S-Fig. 16 Solid and fluid domain of Gyroid TPMS structure.**

**S-Table 13 Pressure differences between the inlet and outlet for different mesh number parameters.**

| Minimum node size (mm) | Mesh numbers | Pressure difference (Pa) |
| --- | --- | --- |
| 0.0030 | 68773 | 9.18 |
| 0.0028 | 88584 | 9.14 |
| 0.0026 | 110639 | 9.12 |
| 0.0024 | 140668 | 9.02 |
| 0.0023 | 159825 | 8.93 |
| 0.0022 | 182625 | 8.93 |
| 0.0021 | 209977 | 8.94 |
| 0.0020 | 243074 | 8.92 |

**S-Table 14 Boundary conditions used in simulation.**

| Conditions | Value |
| --- | --- |
| Hot fluid inlet velocity | 0.1 m/s |
| Cold fluid inlet velocity | 0.1 m/s |
| Hot fluid inlet temperature | 30℃ |
| Cold fluid inlet temperature | 20℃ |
| Outlet pressure | 0 Pa |

Building on the experiment-free screening framework in Eqs. 1–2, we further asked whether the same sub-unit-cell descriptors can quantitatively explain the measured transport performance. Using all 42 data points (7 Reynolds numbers × 6 TPMS architectures), we performed a log–linear regression in a classical transport scaling form, treating j/f as the response and the four intrinsic descriptors as predictors. The resulting fit,

| ${(j/f)}_{\text{quant}}=0.0265{(Re)}^{0.276}{(\frac{S}{V^{2/3}})}^{4.92}{(\frac{d}{\sigma})}^{0.51}N^{0.20}\mid\chi\mid^{-0.96}$ | **(13)** |
| --- | --- |

achieves a high goodness of fit (R^2^=0.89), providing an independent, performance-based validation that the descriptor set used in Eq. 1 captures the dominant geometric contributions governing transport.

Importantly, this quantitative regression is consistent with the hierarchy revealed by the global sensitivity and PCA analyses (Figs. 5–6), which identify uniformity and conduit density as the two most influential drivers. Motivated by this mechanistic dominance, we further tested a parsimonious reduced scaling using the condensed descriptor $\bar{d}/\sigma\times N$ from Eq. 2, yielding

| ${(j/f)}_{\text{quant}}^{'}=0.21{(Re)}^{0.276}{(\bar{d}/\sigma\times N)}^{0.30}$ | **(14)** |
| --- | --- |

with R^2^=0.75. Despite its simplicity, this reduced form preserves the correct cross-topology performance ranking, confirming that $\bar{d}/\sigma\times N$ serves as a compact, physically grounded proxy linking the sub-unit-cell decoding to the measured j/f trends.

**S-Fig. 17** Parity plots comparing predicted and simulated $j/f$values for six TPMS architectures across multiple Reynolds numbers. **a:** full descriptor model (Supplementary Eq. 6). **b:** reduced model based on $(d/\sigma)\times N$ (Supplementary Eq. 7). Marker shapes denote TPMS topology, and colors indicate Reynolds number. The dashed line represents perfect agreement. Both models reproduce the correct performance ranking and order of magnitude, with the full model exhibiting improved quantitative accuracy.

## 4.2 The experimental analysis

**S-Fig. 18**, **S-Table 15**, **S-Table 16** and **S-Table 18** describe the experimental procedure for the liquid-cooling test.

The heat transfer performance of both hot and cold side was calculated by the following equation: *Q* = *m*∙c*_p_*∙Δ*T*, where *m* is the mass flow rate of the fluid, *c_p_* is specific heat capacity at constant pressure, and Δ*T* is the temperature difference between the outlet and inlet*.* Then the heat transfer performance of the system can be obtained by: $\frac{\text{Q}_{\text{h}\text{ot}}\text{+}\text{Q}_{\text{cold}}}{\text{2}}$, where *Q*_hot_, *Q*_cold_ are the heat transfer performance for the hot and cold side, respectively.

Measurement uncertainties for temperature, pressure, and flow rate, evaluated via the Kline‑McClintock method ^[98]^, are provided in **S-Table 18**. The resulting propagated uncertainties for the derived quantities are 2.3 % for the heat‑transfer rate, 2.6 % for the flow resistance, 4.4 % for the *j*‑factor, and 4.9 % for the *f*‑factor. Besides, the pressure sensor uncertainty is 0.2% of full scale (0.3 MPa), corresponding to an absolute uncertainty of 0.6 kPa and relative errors of ~2% at 30 kPa and ~0.48% at 125 kPa (Re = 2970), well within acceptable limits.

**S-Fig. 18 The schematic diagram of the heat exchange experiment bench.**

**S-Table 15 System components for heat exchange experiment bench.**

| Components | Information |
| --- | --- |
| Thermostatic water bath | Adjustable between water temperature  0~50 ℃, volume flow 0~30 L/min |
| Water pump | 120 W, 0~10 L/min |
| Water valve | On/off ball valve |
| Data logger | Agilent 34927A (0.004% dcV accuracy) |

**S-Table 16 Experimental conditions.**

| Fluid type | Fluid 1 (hot) | Fluid 2 (cold) |
| --- | --- | --- |
|  | 50% ethylene glycol | water |
| Inlet temperature (℃) | $\sim$35 | ambient temperature |
| Volume flow rate (L min^-1^) | 10 | 2.5 |

**S-Table 17 The uncertainty analysis in the experiment.**

| Parameters | Measuring instruments | Range | Max. uncertainties |
| --- | --- | --- | --- |
| Water volume flow | Electromagnetic flowmeter | 0-30 L min^-1^ | ±0.15% (Reading) |
| Temperature | T type thermocouple | 0-200 ℃ | ±0.1℃ |
| Pressure | Absolute pressure sensor | 0-300 kPa | ±0.12% Full scale |

**S-Table 18 Inlet and outlet temperatures of the hot and cold fluids for the TPMS heat exchangers.**

| TPMS heat exchangers | V_cold_ | T_in,cold_ | T_out,cold_ | Q_cold_ | V_hot_ | T_in,hot_ | T_out,hot_ | Q_hot_ |
| --- | --- | --- | --- | --- | --- | --- | --- | --- |
|  | L/min | ℃ | ℃ | W | L/min | ℃ | ℃ | W |
| Primitive | 2.5 | 16.5 | 26.0 | 1670.0 | 10 | 34.9 | 32.0 | 1736.8 |
| F-RD | 2.5 | 16.6 | 23.9 | 1281.0 | 10 | 35.0 | 32.8 | 1332.2 |
| Gyroid | 2.5 | 16.7 | 27.5 | 1893.0 | 10 | 35.1 | 31.9 | 1968.7 |
| Fischer-Koch | 2.5 | 16.4 | 28.2 | 2073.0 | 10 | 35.2 | 31.6 | 2155.9 |
| V_cold_ and V_hot_ : Volumetric flow rates of the cold and hot streams, respectively. T_in, cold_ and T_out, cold_ : Inlet and outlet temperatures of the cold stream. T_in, hot_ and T_out, hot_ : Inlet and outlet temperatures of the hot stream. Q_cold_ and Q_hot_ :Heat transfer rates calculated from the cold and hot sides. | | | | | | | | |

# 5. Reference

1 W. Fokke, J. Zachаriades, J. Olivier, The effect of the corrugation inclination angle on the thermo hydraulic performance of plate heat exchangers–Chemical Engineering Research Group, 30 Jan. 1985, Pretoria, Per. of South Africa.

2 B.O. Hasan, Turbulent Prandtl number and its use in prediction of heat transfer coefficient for liquids, Al-Nahrain Journal for Engineering Sciences 10(1) (2007) 53-64.

3 D.A. Nield, A. Bejan, Convection in porous media, Springer2006.

4 R.K. Shah, D.P. Sekulic, Fundamentals of heat exchanger design, John Wiley & Sons2003.

5 A.P. Colburn, A method of correlating forced convection heat-transfer data and a comparison with fluid friction, International Journal of Heat and Mass Transfer 7(12) (1964) 1359-1384.

6 F. ww, Turbulent convective transfer in plate heat exchangers, Int Commun Heat Mass Transf 10 (1983) 201-210.

7 M.R. Muley A, Enhanced heat transfer characteristics of single-phase flows in a plate heat exchanger with mixed chevron plates, Enhanc Heat Transf 4 (1997) 187-201.

8 M.R. Muley A, Metwally HM, Enhanced heat transfer characteristics of viscous liquid flows in a chevron plate heat exchanger, J Heat Transf 121 (1999) 1011-1017.

9 G. Gerd, Volker, Kottke Effects of Wavelength and Inclination Angle on the Homogeneity of Local Heat Transfer Coefficients in Plate Heat Exchangers, Heat Transfer Conference 6 (1998) 203-208.

10 H.M. Li Y, Hu EJ, Experimental investigation of heat transfer and pressure drop in cross-corrugated plate heat exchangers, Proceedings international conference energy Environ. Shanghai, China (1998) 288-293.

11 H. M, An experimental investigation of the performance of cross-corrugated plate heat exchangers, J Enhanc Heat Transf 10 (2003) 379-393.

12 H.C. Blomerius H, Mitra NK, Numerical investigation of ﬂow ﬁeld and heat transfer in cross-corrugated ducts, J Heat Transf 121 (1999) 314-321.

13 S.C. Lee YS, Sun YM, Ye JC, Experimental study in heat transfer in wavy channels, J Enhanc Heat Transf 12 (2003) 21–29.

14 H.C. Lin JH, Su CC, Dimensional analysis for the heat transfer characteristics in the corrugated channels of plate heat exchangers, Int Commun Heat Mass Transf 34 (2007) 304-312.

15 D. Svaic, Influence of chevron plates geometry on performances of plate heat exchangers, Teh Vjesn 14 (2007) 37-45.

16 M.A. Kanaris AG, Paras sv, Optimal design of a plate heat exchanger with undulated surfaces, Int J Therm Sci 48 (2009) 1184-1195.

17 K.M. Khan TS, Chyu MC, Ayub ZH, Experimental investigation of single phase convective heat transfer coefficient in a corrugated plate heat exchanger for multiple plate configurations., Appl Therm Eng 30 (2010) 1058-1065.

18 Z.D.J. Wang Q W, Xie G N, Experimental study and genetic-algorithm-based correlation on pressure drop and heat transfer performances of a cross-corrugated primary surface heat exchanger, J Heat Transf 131 (2009) 61-82.

19 D.W. Guo CS, Cheng L, Characteristics of heat transfer and resistance of double chevron plate heat exchanges with diﬀerent corrugation pitch, Adv Intell Soft Comput AISC 143 (2012) 169–174.

20 D.S. Shaji K, Eﬀect of plate characteristics on axial dispersion and heat transfer in plate heat exchangers, J Heat Transf 135 (2013) 41-48.

21 W.Y. Zhao YH, Cheng HJ, Zhu GL, Numerical simulation of corrugated depth on the performance of plate heat exchanger, Adv Mater Res 860 (2013) 696–699.

22 S.K. Han w, Aute V, Ding G, Hwang Y, Radermacher R., Numerical simulation and optimization of single-phase turbulent flow in chevron-type plate heat exchanger with sinusoidal corrugations, HVAC&R Res 17 (2011) 186-197.

23 L.K. Lee J, Friction and Colburn factor correlations and shape optimization of chevron-type plate heat exchangers, Appl Therm Eng 89 (2015) 62-69.

24 L.K. Lee J, Flow characteristics and thermal performance in chevron type plate heat exchangers, Int J Heat Mass Transf 78 (2014) 699-706.

25 L.K. Han DH, Kim YH, The characteristics of condensation brazed plate heat exchangers with different chevron angles, J Korean Phys Soc 43 (2003) 66-73.

26 O.N. Wurfel R, Experimental investigations of heat transfer and pressure drop during the condensation process within plate heat exchangers of the her-ringbone-type, Int J Therm Sci 43 (2004) 59-68.

27 K.S. Djordjevic E, Flow boiling of R134a and ammonia in a plate heat exchanger, Int J Heat Mass Transf 51 (2008) 6235-6242.

28 J.A. Hayes N, Ayub ZH, Study of carbon dioxide condensation in chevron plate exchangers; heat transfer analysis, Int J Heat Mass Transf 54 (2011) 1121-1131.

29 J.A. Hayes N, Ayub ZH, Study of carbon dioxide condensation in chevron plate exchangers; pressure drop analysis, Int J Heat Mass Transf 55 (2012) 2916-2925.

30 S.T. Huang J , Bailey-Mcewan M, Heat transfer and pressure drop in plate heat exchanger refrigerant evaporators, Int J Refrig 35 (2012) 325-335.

31 K.T. Khan MS, Chyu MC, Ayub ZH, Evaporation heat transfer and pressure drop of ammonia in a mixed conﬁguration chevron plate heat exchanger, Int J Refrig 41 (2014) 92–102.

32 K.S. Müller A, The experimental determination of heat transfer and pressure drop during condensation in a plate heat exchanger with corrugated plates, WIT Trans Eng Sci 83 (2014) 337–349.

33 U.M. Imran M, Yang Y, Park BS, Flow boiling of R245fa in the brazed plate heat exchanger: thermal and hydraulic performance assessment, Int J Heat Mass Transf 110 (2017) 657–670.

34 L.D. Kim D, Jang DS, Jeon Y, Kim Y, Comparative evaluation of ﬂow boiling heat transfer characteristics of R1234ze(E) and R134a in plate heat exchangers with diﬀerent Chevron angles, Appl Therm Eng 132 (2018) 719–729.

35 M.H. Miyata K, Taniguchi T, Umezawa S, Sugita K, Eﬀect of the Chevron angle on cooling heat transfer characteristics of supercritical pressure ﬂuids in plate heat exchangers, Heat Transf Eng 0 (2018) 1-16.

36 K. Yeranee, Y. Rao, A review of recent investigations on flow and heat transfer enhancement in cooling channels embedded with triply periodic minimal surfaces (TPMS), Energies 15(23) (2022) 8994.

37 K. Dutkowski, M. Kruzel, K. Rokosz, Review of the state-of-the-art uses of minimal surfaces in heat transfer, Energies 15(21) (2022) 7994.

38 M.I. Hassan Ali, O. Al-Ketan, A. Alhammadi, M. Khalil, K. Khan, R.K. Abu Al-Rub, Heat Transfer Characterization of 3D Printable Architected Heat Sinks, ASME 2019 International Mechanical Engineering Congress and Exposition, 2019.

39 M.I. Hassan Ali, O. Al-Ketan, N. Baobaid, K. Khan, R.K. Abu Al-Rub, A Study on the Fluid Flow and Heat Transfer for a Porous Architected Heat Sink Using the Idea of CFD Modelling, ASME 2019 International Mechanical Engineering Congress and Exposition, 2019.

40 W. Li, G. Yu, Z. Yu, Bioinspired heat exchangers based on triply periodic minimal surfaces for supercritical CO2 cycles, Applied Thermal Engineering 179 (2020) 115686.

41 Z.A. Qureshi, E. Elnajjar, O. Al-Ketan, R.A. Al-Rub, S.B. Al-Omari, Heat transfer performance of a finned metal foam-phase change material (FMF-PCM) system incorporating triply periodic minimal surfaces (TPMS), International Journal of Heat and Mass Transfer 170 (2021) 121001.

42 O. Al-Ketan, M. Ali, M. Khalil, R. Rowshan, K.A. Khan, R.K. Abu Al-Rub, Forced Convection Computational Fluid Dynamics Analysis of Architected and Three-Dimensional Printable Heat Sinks Based on Triply Periodic Minimal Surfaces, Journal of Thermal Science and Engineering Applications 13(2) (2020).

43 R. Attarzadeh, M. Rovira, C. Duwig, Design analysis of the “Schwartz D” based heat exchanger: A numerical study, International Journal of Heat and Mass Transfer 177 (2021) 121415.

44 N. Baobaid, M.I. Ali, K.A. Khan, R.K. Abu Al-Rub, Fluid flow and heat transfer of porous TPMS architected heat sinks in free convection environment, Case Studies in Thermal Engineering 33 (2022) 101944.

45 M. Asif, C.A. Grande, TPMS Contactors Designed with Imprinted Porosity: Numerical Evaluation of Momentum and Energy Transport, Industrial & Engineering Chemistry Research 61(50) (2022) 18556-18566.

46 L. Lesmana, M. Aziz, Mechanical Behaviour and Fluid Dynamics Analysis of Metal Hydride for Hydrogen Storage Based on Triply Periodic Minimal Surface Structure, Chemical Engineering Transactions 94 (2022) 901-906.

47 W. Li, W. Li, Z. Yu, Heat transfer enhancement of water-cooled triply periodic minimal surface heat exchangers, Applied Thermal Engineering 217 (2022) 119198.

48 K. Yeranee, Y. Rao, L. Yang, H. Li, Improved Thermal Performance of a Serpentine Cooling Channel by Topology Optimization Infilled with Triply Periodic Minimal Surfaces, Energies 15 (2022).

49 E. Gurra, M. Iasiello, V. Naso, W. Chiu, Numerical Prediction and Correlations of Effective Thermal Conductivity in a Drilled-Hollow-Sphere Architected Foam, Journal of Thermal Science and Engineering Applications 15 (2022) 1-23.

50 S. Rathore, B. Mehta, P. Kumar, M. Asfer, Flow Characterization in Triply-Periodic-Minimal-Surface (TPMS) based Porous Geometries: Part 1 -Hydrodynamics, Transport in Porous Media 146 (2022).

51 J. Wang, K. Chen, M. Zeng, T. Ma, Q. Wang, Z. Cheng, Investigation on flow and heat transfer in various channels based on triply periodic minimal surfaces (TPMS), Energy Conversion and Management 283 (2023) 116955.

52 J. Iyer, T. Moore, D. Nguyen, P. Roy, J. Stolaroff, Heat transfer and pressure drop characteristics of heat exchangers based on triply periodic minimal and periodic nodal surfaces, Applied Thermal Engineering 209 (2022) 118192.

53 M.G. Gado, S. Ookawara, H. Hassan, Utilization of triply periodic minimal surfaces for performance enhancement of adsorption cooling systems: Computational fluid dynamics analysis, Energy Conversion and Management 277 (2023) 116657.

54 K. Yan, J. Wang, L.a. Li, H. Deng, Numerical investigation into thermo-hydraulic characteristics and mixing performance of triply periodic minimal surface-structured heat exchangers, Applied Thermal Engineering 230 (2023) 120748.

55 H. Peng, F. Gao, W. Hu, Design, modeling and characterization on triply periodic minimal surface heat exchangers with additive manufacturing, (2019).

56 M. Alteneiji, M.I.H. Ali, K.A. Khan, R.K.A. Al-Rub, Heat transfer effectiveness characteristics maps for additively manufactured TPMS compact heat exchangers, Energy Storage and Saving 1(3) (2022) 153-161.

57 Z. Cheng, X. Li, R. Xu, P. Jiang, Investigations on porous media customized by triply periodic minimal surface: Heat transfer correlations and strength performance, International Communications in Heat and Mass Transfer 129 (2021) 105713.

58 T. Femmer, A.J. Kuehne, M. Wessling, Estimation of the structure dependent performance of 3-D rapid prototyped membranes, Chemical Engineering Journal 273 (2015) 438-445.

59 T. Dixit, E. Al-Hajri, M.C. Paul, P. Nithiarasu, S. Kumar, High performance, microarchitected, compact heat exchanger enabled by 3D printing, Applied Thermal Engineering 210 (2022) 118339.

60 L.K. Dharmalingam, V. Aute, J. Ling, Review of triply periodic minimal surface (TPMS) based heat exchanger designs, (2022).

61 D. Clarke, P. Galvosas, D. Holland, Characterization of unsteady flow in a 3D ‐printed Schwarz Diamond monolith using magnetic resonance velocimetry, AIChE Journal (2023).

62 L.A. Lesmana, C. Lu, F. Chen, M. Aziz, Triply periodic minimal surface gyroid structure as effective metal hydride hydrogen storage reactor: Experimental study, Thermal Science and Engineering Progress 42 (2023) 101903.

63 B.W. Reynolds, C.J. Fee, K.R. Morison, D.J. Holland, Characterisation of Heat Transfer within 3D Printed TPMS Heat Exchangers, International Journal of Heat and Mass Transfer 212 (2023) 124264.

64 M.I.H. Ali, O. Al-Ketan, M. Khalil, N. Baobaid, K. Khan, R.K. Abu Al-Rub, 3D Printed Architected Heat Sinks Cooling Performance in Free and Forced Convection Environments, ASME 2020 Heat Transfer Summer Conference collocated with the ASME 2020 Fluids Engineering Division Summer Meeting and the ASME 2020 18th International Conference on Nanochannels, Microchannels, and Minichannels, 2020.

65 J. Kim, D.-J. Yoo, 3D printed compact heat exchangers with mathematically defined core structures, Journal of Computational Design and Engineering 7(4) (2020) 527-550.

66 D.A. Clarke, F. Dolamore, C.J. Fee, P. Galvosas, D.J. Holland, Investigation of flow through triply periodic minimal surface-structured porous media using MRI and CFD, Chemical Engineering Science 231 (2021) 116264.

67 Z. Cheng, R. Xu, P.-X. Jiang, Morphology, flow and heat transfer in triply periodic minimal surface based porous structures, International Journal of Heat and Mass Transfer 170 (2021) 120902.

68 R. Attarzadeh, S.-H. Attarzadeh-Niaki, C. Duwig, Multi-objective optimization of TPMS-based heat exchangers for low-temperature waste heat recovery, Applied Thermal Engineering 212 (2022) 118448.

69 M. Khalil, M.I. Hassan Ali, K.A. Khan, R. Abu Al-Rub, Forced convection heat transfer in heat sinks with topologies based on triply periodic minimal surfaces, Case Studies in Thermal Engineering 38 (2022) 102313.

70 D. Mahmoud, S. Tandel, M. Yakout, M. Elbestawi, F. Mattiello, S. Paradiso, C. Ching, M. Zaher, M. Abdelnabi, Enhancement of heat exchanger performance using additive manufacturing of gyroid lattice structures, The International Journal of Advanced Manufacturing Technology 126 (2023).

71 W. Tang, H. Zhou, Y. Zeng, M. Yan, C. Jiang, P. Yang, Q. Li, Z. Li, J. Fu, Y. Huang, Y. Zhao, Analysis on the convective heat transfer process and performance evaluation of Triply Periodic Minimal Surface (TPMS) based on Diamond, Gyroid and Iwp, International Journal of Heat and Mass Transfer 201 (2023) 123642.

72 D. Liang, C. Shi, W. Li, W. Chen, M.K. Chyu, Design, flow characteristics and performance evaluation of bioinspired heat exchangers based on triply periodic minimal surfaces, International Journal of Heat and Mass Transfer 201 (2023) 123620.

73 J.D. Shiming Gao, Shuo Qu, Hui Liu, Xu Song, Numerical and experimental investigation of additively manufactured shell-lattice copper heat exchanger, International Communications in Heat and Mass Transfer 147 (2023) 106976.

74 S. Gao, S. Qu, J. Ding, H. Liu, X. Song, Influence of cell size and its gradient on thermo-hydraulic characteristics of triply periodic minimal surface heat exchangers, Applied Thermal Engineering 232 (2023) 121098.

75 J. Zhang, Z. Wang, X. Wang, H. Tan, G. Lisak, Numerical study on biomass co-firing with coal in a pilot-scale pressurized oxy-fuel combustor, Fuel 376 (2024) 132672.

76 X. Zhang, Z. Zhang, S. Li, G. Yan, H. Li, H. Dong, M. Sun, Y. Zhang, Y. Song, Enhanced melting behavior of phase change materials using anisotropic Primitive sheet-networks triply periodic minimal surface structure, Energy (2025) 136624.

77 M. Sun, Y. Liang, X. Jiang, Y. Song, Flow and heat transfer characteristics of anisotropic Kelvin cells: Influence of stretching ratio and mechanistic analysis, International Communications in Heat and Mass Transfer 169 (2025) 109896.

78 X. Zhang, Z. Zhang, S. Li, G. Yan, H. Li, H. Dong, M. Sun, Y. Zhang, Y. Song, Enhanced melting behavior of phase change materials using anisotropic Primitive sheet-networks triply periodic minimal surface structure, Energy 328(136624) (2025).

79 T. Si, K. Sun, H. Zhang, Q. Wang, Z. Cheng, Lattice-scale topology optimization of TPMS structure for heat transfer., International Journal of Heat and Mass Transfer 251(127288) (2025).

80 T. Si, H. Zhang, Q. Wang, Z. Cheng, Improvement of Heat Conduction in Triply Periodic Minimal Surface HeatSinks Filled with Phase Change Materials. , Heat Transfer Engineering 47(2) (2026) 182–192.

81 K. Yan, H. Deng, Y. Wu, T. Yu, Y. Xiao, J. Wang, Gyroid-structured heat exchanger optimization via lattice geometric manipulation for enhanced thermo-hydraulic performance: an experimental and numerical research, International Journal of Thermal Sciences 215(109966) (2025).

82 K. Yan, H. Deng, Y. Wu, J. Wang, Y. Huang, Effects of lattice geometric manipulation on thermo-hydraulic performance of Gyroid-structured heat exchanger: A numerical study, International Journal of Heat and Mass Transfer 248(127217) (2025).

83 H. Yang, Z. Wang, R. Bao, B. Zhang, X. Zhu, H. Wang, A novel hybrid battery thermal management system using TPMS structure and delayed cooling scheme, Applied Thermal Engineering 259(124901) (2025).

84 J. Sun, X. Li, H. Mao, Y. M, J. Liu, X. Chen, Numerical analysis of the mechanism of porosity effect on the thermal–hydraulic performance of Gyroid-type TPMS structures in combined aero engines, Applied Thermal Engineering 264(125453) (2025).

85 Z. Men, W. Chen, Q. Li, S. Liu, Topology optimization of the IWP triply periodic minimal surfaces (TPMS) heat sink based on porous media effective model, International Journal of Heat and Mass Transfer 240(126657) (2025).

86 W.S. Huang, H.Y. Ning, G.H. Tang, Investigation of high-compactness and high-efficiency TPMS precoolers for precooled aero-engine, Energy 319(135014) (2025).

87 J. Chen, X. Liu, Y. Li, X. Feng, J. Chen, H. Zhu, W.-Q. Tao, Numerical and experimental investigation of TPMS-structured cold plates for electronic device cooling, Applied Energy 401(126745) (2025).

88 S.T. Hyde, L. de Campo, C. Oguey, Tricontinuous mesophases of balanced three-arm ‘star polyphiles’, Soft Matter 5(14) (2009).

89 H.Z. Zhong, T. Song, C.W. Li, R. Das, J.F. Gu, M. Qian, The Gibson-Ashby Model for Additively Manufactured Metal Lattice Materials: Its Theoretical Basis, Limitations and New Insights from Remedies, Current Opinion in Solid State and Materials Science 27 (2023) 101081.

90 H.Z. Zhong, R. Das, J. Gu, M. Qian, Low-density, high-strength metal mechanical metamaterials beyond the Gibson-Ashby model, Materials Today 68 (2023) 96-107.

91 Interactive Voronoi Diagram <<http://sygreer.com/projects/voronoi/>>.

92 D. Liang, K. Yang, H. Gu, W. Chen, M.K. Chyu, The effect of unit size on the flow and heat transfer performance of the “Schwartz-D” heat exchanger, International Journal of Heat and Mass Transfer 214 (2023) 124367.

93 B.W. Reynolds, Simulation of flow and heat transfer in 3D printable triply periodic minimal surface heat exchangers, (2020).

94 P. Knödler, V. Dreissigacker, Fluid dynamic assessment and development of Nusselt correlations for Fischer Koch S structures, Energies 17(3) (2024) 688.

95 J. Wang, C. Qian, X. Qiu, B. Yu, L. Yan, J. Shi, J. Chen, Numerical and experimental investigation of additive manufactured heat exchanger using triply periodic minimal surfaces (TPMS), Thermal Science and Engineering Progress 55 (2024) 103007.

96 S. Samson, P. Tran, P. Marzocca, Design and modelling of porous gyroid heatsinks: Influences of cell size, porosity and material variation, Applied Thermal Engineering 235 (2023) 121296.

97 K. Ali Abd Al-Hameed, Spearman's correlation coefficient in statistical analysis, International Journal of Nonlinear Analysis and Applications 13(1) (2022) 3249-3255.

98 S.J. Kline, The purposes of uncertainty analysis, (1985).
